# Poliovirus 3D^pol^ polymerase region is essential for cleavage of poliovirus 3AB *in vivo*

**DOI:** 10.1371/journal.ppat.1014241

**Published:** 2026-05-13

**Authors:** Minetaro Arita

**Affiliations:** Department of Virology II, National Institute of Infectious Diseases, Japan Institute for Health Security, Gakuen, Musashimurayama-shi, Tokyo, Japan; University of Maryland at College Park: University of Maryland, UNITED STATES OF AMERICA

## Abstract

Poliovirus (PV) genome encodes a large single polyprotein that is processed by viral proteases to form an active replication complex through either *cis* or *trans* interactions between the viral proteins (*i.e.*, interactions between viral proteins encoded on the same polyprotein molecule or between those encoded on different polyprotein molecules, respectively). In the processing of polyprotein, the cleavage of viral 3AB into 3A and 3B is unique, as it requires host factors (PI4KB/OSBP) and viral protease (3C^pro^/3CD^pro^) in cultured cells (*i.e.*, *in vivo*). Here, we show viral/host requirements for the cleavage of 3AB *in vivo*. In a polyprotein encoding 2BC3ABCD of PV, cleavage of 3AB requires the activity of PI4KB as well as the entire 3D^pol^ region; even a partial deletion of the 3D^pol^ region severely affects the cleavage in the polyprotein. The activity of OSBP and the binding activity of 3CD^pro^ to negatively charged molecules are not required for the cleavage in the polyprotein. PV mutants with premature termination codons or in-frame deletions in the 3D^pol^-coding region are generally quasi-infectious in *trans*-rescued replication with 3CD^pro^, causing extensive in-frame genome duplication or deletion. Surprisingly, some PV mutants lacking the C-terminal peptides of 3D^pol^ showed stable replication without any reversion in the presence of 3CD^pro^ provided in *trans*; 3D^pol^ provided in *trans* could rescue the defect in 3AB cleavage via amino acid residues involved in 3D^pol^-3AB and 3D^pol^-3D^pol^ interactions, indicating a remarkable overlap with those required for the uridylylation of 3B. This work reveals novel roles of the 3D^pol^ region, offering insights into the polyprotein processing and recombination.

## Author summary

Among the cleavages of the polyprotein of enteroviruses, including poliovirus, cleavage of 3AB into 3A and 3B is unique, as it requires both host factors (PI4KB/OSBP) and viral proteases. Moreover, the 3AB cleavage requires active viral proteases (3C^pro^/3CD^pro^) encoded on the same polyprotein molecule (provided in *cis*). The present study reveals that cleavage of 3AB requires host PI4KB activity and, unexpectedly, the 3D^pol^ region of the polyprotein; the defect in 3AB cleavage in polyprotein variants lacking partial 3D^pol^ regions can be rescued by exogenously expressed 3D^pol^ (provided in *trans*). Amino acid residues required for the 3D^pol^-3AB and 3D^pol^-3D^pol^ interactions, most of which are also required for the uridylylation of 3B, are critical in the cleavage of 3AB. These results suggest that 3AB cleavage requires protease activity in *cis*, but the efficiency of cleavage is strongly affected by *trans*-acting viral/host proteins and cellular context, suggesting a hybrid mechanism. In addition, a novel role of the 3D^pol^-coding region in the dynamic reconstruction of viral genome structure was also discovered during this study. Why such a complex mechanism has evolved in the cleavage of enterovirus 3AB protein remains an open question.

## Introduction

Picornavirus is a small non-enveloped virus with a positive-sense single-stranded RNA genome of about 7,500 nt, including poliovirus (PV) as the typical member of this family (*Enterovirus coxsackiepol* species [previously called *Enterovirus C* species], the genus *Enterovirus,* the family *Picornaviridae*) [1]. The genome of PV encodes a single large polyprotein (about 2,200 amino acids [aa]) that is subsequently processed into each viral protein. The polyprotein is initially processed into three precursor proteins, P1 (coding VP4VP2VP3VP1), P2 (coding 2A2B2C), and P3 (coding 3A3B3C3D), by viral proteases (2A^pro^ and 3C^pro^/3CD^pro^/3ABC^pro^) [2–4]. P1 is further processed into each viral capsid protein, P2 is processed into proteins that have roles in viral RNA synthesis and in virion production/release (2A^pro^ protease, 2B viroporin, 2C^ATPase/hel^ ATPase/helicase) [2,5–9], and P3 is processed into proteins that most directly serve for the RNA synthesis (3A [unknown enzymatic function/recruitment of host proteins GBF1/ACBD3/PI4KB], 3B [also known as VPg, the primer for RNA synthesis], 3C^pro^ protease, 3D^pol^ polymerase) [1,10–17]. Processing intermediates produced during the processing (*i.e.*, 2BC, 3AB, 3CD^pro^, etc.) play critical roles in replication and virion production as well as the fully processed viral proteins [3,18–25]; no single disruption of viral protein [26], except for 2A^pro^ [27], or of processing intermediate [28] allows replication.

Processing of the polyprotein is controlled in *cis* cleavage (*i.e.*, cleavage of the polyprotein by viral proteases [2A^pro^/3C^pro^/3CD^pro^/3ABC^pro^], which are encoded in the target polyprotein molecule itself, thus authentic self-cleavage) and in *trans* cleavage (*i.e.*, cleavage of polyprotein by the proteases, which are encoded in polyprotein molecules other than the target polyprotein). Processing of P1, which is conducted by 3CD^pro^ [3,22], occurs in *trans* [29]. Disruptions of the polyprotein synthesis [28] or introduction of mutations in the P2 or P3 regions, which do not affect the protease activity, cause aberrant processing and lethality of the virus [30–34], underscoring the structural integrity of polyprotein precursors for the processing. Replication of a PV mutant that encodes inactive 3CD^pro^ could not be rescued by active 3CD^pro^ provided in *trans,* indicating the essential *cis* role of 3CD^pro^ [26].

Cleavage of 3AB into 3A and 3B is unique in polyprotein processing *in vivo* (*i.e.*, in cultured cells), as it requires both host PI4KB/OSBP pathway and active viral 3C^pro^/3CD^pro^ provided in *cis* [16,26,35–38]; 3AB could be cleaved out from a PV polyprotein that encodes inactive 3C^pro^/3CD^pro^ by active 3CD^pro^ provided in *trans*, however further processing of 3AB into 3A and 3B did not occur [26]. PI4KB is required for the early phase of replication [39,40], suggesting the importance of 3AB cleavage during this phase. *In vitro* (*i.e.*, in a cell-free system), only the membrane-associated 3AB could be cleaved by 3C^pro^/3CD^pro^ [20], indicating the importance of the interaction between 3AB and lipids. PI4KB is a phosphatidylinositol-4 kinase that produces phosphatidylinositol 4-phosphate (PI4P) mainly at the Golgi [41], and serves as a host factor for enterovirus (EV) replication [16]. OSBP is a sterol/PI4P transporter that transfers cholesterol between the endoplasmic reticulum and the *trans* Golgi in a PI4P-dependent manner and contributes to the homeostasis of cholesterol and lipid [42–46] and also serves as a host factor for EV replication [35,47,48]. A functional link between PI4KB and OSBP in PV replication was revealed by common resistance mutations in 3A and 2B to PI4KB/OSBP inhibitors [40,49,50], PI4KB-dependent localization of OSBP to viral replication organelle (RO) and accumulation of cholesterol on the RO [51]. In a current model of EV replication, PI4KB is activated by the viral proteins (2C^ATPase/hel^, 2BC, 3AB, 3CD^pro^, and 3D^pol^) and provides PI4P to recruit OSBP, then OSBP accumulates cholesterol to facilitate cleavage of 3AB and the formation of RO for viral plus-strand RNA synthesis [36–40,47,50–53]. The mechanistic link between 3AB cleavage and the development of RO remains unknown [50]. There is a high genetic barrier to the independence of PV replication from this host pathway [54], indicating a strong dependence of EV replication on it and highlighting the enigmatic properties of 3AB cleavage, which evolved under uncharacterized selection pressure.

Here, we have analyzed viral and host factors required for the cleavage of 3AB into 3A and 3B *in vivo*. Cleavage of 3AB in a polyprotein encoding PV 2BC3ABCD requires the activity of PI4KB, but not that of OSBP. The entire 3D^pol^ region of the polyprotein is required for the cleavage of 3AB; the role of the 3D^pol^ region could be complemented by 3CD^pro^ or 3D^pol^ provided in *trans* via 3D^pol^-3AB and 3D^pol^-3D^pol^ interactions. A role of the 3D^pol^-coding region in the dynamic reconstruction of viral genome structure has also been shown.

## Results

### The *cis* role of 3CD^pro^ in PV replication

**Fig 1:**
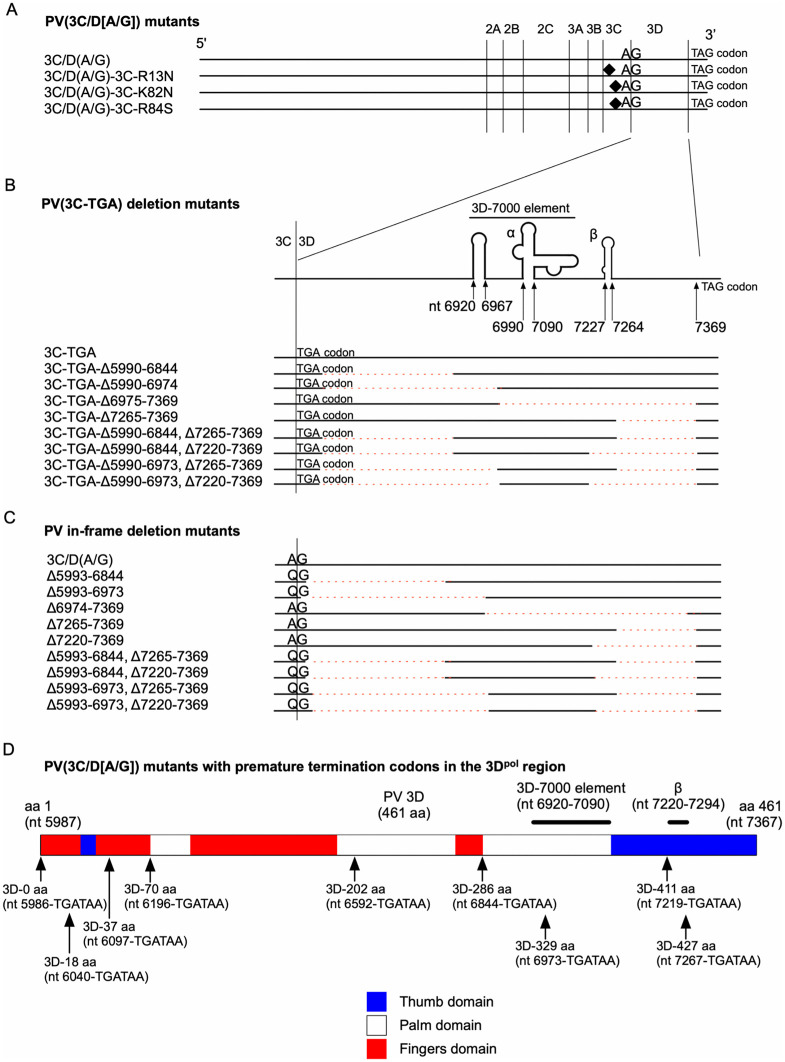
Schematic view of PV replicon mutants used in this study. Introduced amino acid substitutions or deletions are shown. These replicons encode firefly luciferase as a reporter for the replication and can replicate only in the presence of the 3CD^pro^ provided in *trans*. For the analysis of *cis* 3AB cleavage, mutations examined in (A) and (D) were introduced in a PV polyprotein (AG-PV-2B2CP3) expression vector. The positions of the nucleotides are of PV1(Mahoney) genome (GenBank accession number V01149.1). **(A)** The 3C-R13N, 3C-K82N, and 3C-R84S amino acid substitutions, which abolish the binding to viral RNA and phospholipids, were introduced to the PV(3C/D[A/G]) replicon. The PV(3C/D[A/G]) replicon has a disrupted cleavage site between the 3C^pro^ and 3D^pol^. **(B)** Deletions were introduced in the 3D^pol^-coding region of the PV(3C-TGA) replicon. The PV(3C-TGA) replicon has a termination codon (TGA) immediately after the 3C^pro^-coding region. **(C)** In-frame deletions were introduced in the 3D^pol^ coding region of the PV(3C/D[A/G]) replicon. For the mutants with deletions immediately after the 3C^pro^-coding region, the cleavage sites between 3C^pro^ and 3D^pol^ retain the WT sequence (Q/G) to minimize the potential steric hindrance from the partially deleted 3D^pol^ structure. **(D)** Tandem premature termination codons (TGATAA) were introduced in the 3D^pol^-coding region of the PV(3C/D[A/G]) replicon. Location of the protein domains (thumb, palm, and fingers) of the 3D^pol^ is shown [100].

To analyze the *cis* role of the 3CD^pro^ protease and its coding region, *trans*-rescued replication of a total of four sets of PV mutants was analyzed with 3CD^pro^ provided in *trans* [26] (**Fig 1**). 3CD^pro^ provided in *trans* could efficiently rescue a defective PV replicon mutant that has a disrupted cleavage site between 3C^pro^ and 3D^pol^ (3C/D[A/G] mutant) [26]; however, why 3CD^pro^ has to be provided in *trans,* remains to be clarified. For this aim, *trans*-rescued replication of PV mutants that have aa substitutions in the 3C^pro^ region, which affect interaction with negatively charged molecules (viral RNA and phospholipids) (3C-R13N, 3C-K82N, and 3C-R84S) [23,55–57] along with the disrupted cleavage site between 3C^pro^ and 3D^pol^ was analyzed (**Fig 1A**). In the 3D^pol^-coding region, there are RNA structures that are required for the replication in *cis*: α (nt 6995–7069 in PV1[Mahoney] genome) and β (nt 7227–7264 in PV1[Mahoney] genome) [58], and 3D-7000 (nt 6920–7090 in PV1[Mahoney] genome) [59]. To determine the essential 3D^pol^-coding region required for the *trans*-rescued replication, three sets of PV mutants were examined. The first set of PV mutants has a termination codon immediately after the 3C^pro^-coding region, accompanied by deletions in the 3D^pol^-coding region (**Fig 1B**). This set of PV mutants enables the evaluation of the importance of these RNA structures independently of the 3D^pol^ peptides encoded within them. The second set of mutants has deletions that are almost identical to those in the first set of mutants, but in-frame deletions without the termination codon immediately after the 3C^pro^-coding region (**Fig 1C**). This set of mutants enables the evaluation of the importance of the encoded peptides within the RNA structures, thereby complementing the analysis using the first set of mutants. For the mutants that have in-frame deletions immediately after the 3C^pro^-coding region (*e.g.*, Δ5993–6844 mutant), the WT cleavage site between 3C^pro^ and 3D^pol^ was retained to minimize the potential steric hindrance caused by the fused 3D^pol^ peptides. The third set of mutants has premature termination codons in the 3D^pol^-coding region (**Fig 1D**). The insertion of premature termination codons allows evaluation of the importance of the 3D^pol^ peptides independently of the RNA structures in the coding region. To minimize the potential pseudoreversion of the mutants in the introduced premature termination codons, tandem premature termination codons (TGATAA) were introduced in the 3D^pol^-coding region.

**Fig 2:**
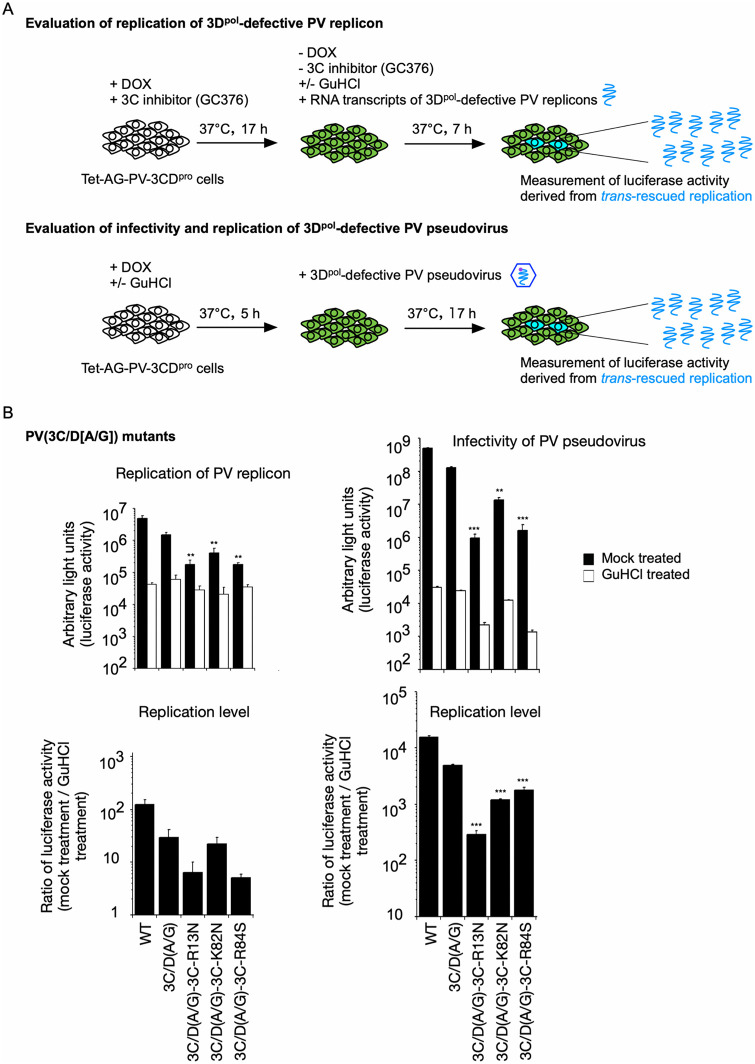
Experimental design for *trans* rescue of replication of PV replicons and infection of PV_pv_. **(A)** Schematic view of the *trans*-rescue experiment. For the replication assay, PV 3CD^pro^ was expressed in the presence of DOX (1 mg/mL) and GC376 (100 μM) at 37°C for 17 h. The RNA transcripts of each PV replicon mutant with a firefly luciferase reporter were transfected into the cells (4 × 10^4^ cells) in the absence of DOX and GC376 and in the presence or absence of GuHCl (a 2C inhibitor, 2 mM). The luciferase signals in the cells were analyzed at 7 h p.t. For the infectivity assay, PV 3CD^pro^ was expressed in the presence of DOX (1 mg/mL) and in the presence or absence of GuHCl (2 mM) at 37°C for 5 h. PV_pv_ mutants with a firefly luciferase reporter were then added to the cells (8 × 10^3^ cells). The luciferase signals in the cells were analyzed at 17 h p.i. **(B)** Replication and infectivity of PV mutants with amino acid substitutions that abolish the binding to viral RNA and phospholipids. The luciferase signals measured at 7 h p.t. of RNA or 17 h p.i. with PV_pv_ are shown. Replication levels are the ratio of the luciferase activities observed in the absence of GuHCl to those observed in the presence of GuHCl. The data represent the mean and standard deviation of three independent experiments with two biological replicates.

These mutations were introduced into the genome of the PV replicon mutant (3C/D[A/G] mutant) with a firefly luciferase reporter [26]. The replicon could express the 3CD^pro^ variants in *cis* but not 3C^pro^ and 3D^pol^, thus lacking polymerase activity and replicating only in the presence of 3CD^pro^ provided in *trans*, which provides 3D^pol^ after the processing [26] (**Fig 2A**). *Trans*-rescued replication of the PV mutants was evaluated by transfection of the RNA transcripts or by infection with PV pseudovirus (PV_pv_) produced from these RNA transcripts. Infectivity of PV_pv_ measured in the presence of GuHCl (a 2C^ATPase/hel^ inhibitor) can be attributed to the initial translation before replication from the viral genome introduced in the cells, thus reflecting the titer of PV_pv_, and that measured in the absence of GuHCl reflects both the titer of PV_pv_ and the replication level of each mutant.

PV mutants with the aa substitutions in the 3C^pro^ region (3C-R13N, 3C-K82N, and 3C-R84S) showed reduced replication levels *in vivo* (*i.e.*, in cultured cells) after RNA transfection. While the 3C-R13N substitution may require additional aa substitutions to efficiently suppress replication *in vitro* (*i.e.*, in a cell-free system) [60], the single 3C-R13N substitution affected *in vivo* PV replication (approximately 1/10 of that of the parental 3C/D[A/G] mutant), or only moderately with the 3C-K82N substitution (**Fig 2B**). In contrast to the relatively moderate effects observed in RNA transfection, the 3C-R13N and 3C-R84S substitutions severely reduced PV_pv_ infectivity *in vivo*, both in the absence and in the presence of GuHCl, suggesting lower titers for these mutants. A relatively moderate effect of the K82N substitution compared with that of the R84S substitution was observed in PV_pv_ infectivity, consistent with a previous report [61]. The replication levels measured by RNA transfection and PV_pv_ infection showed a similar profile, except for the 3C-R84S mutant. Nevertheless, the replication levels of the 3C-R84S mutant were lower than those of the parental 3C/D(A/G) mutant in both systems. These results suggest that 3C^pro^/3CD^pro^ play substantial roles in replication and virion production in *cis,* via interactions with the negatively charged molecules.

**Fig 3:**
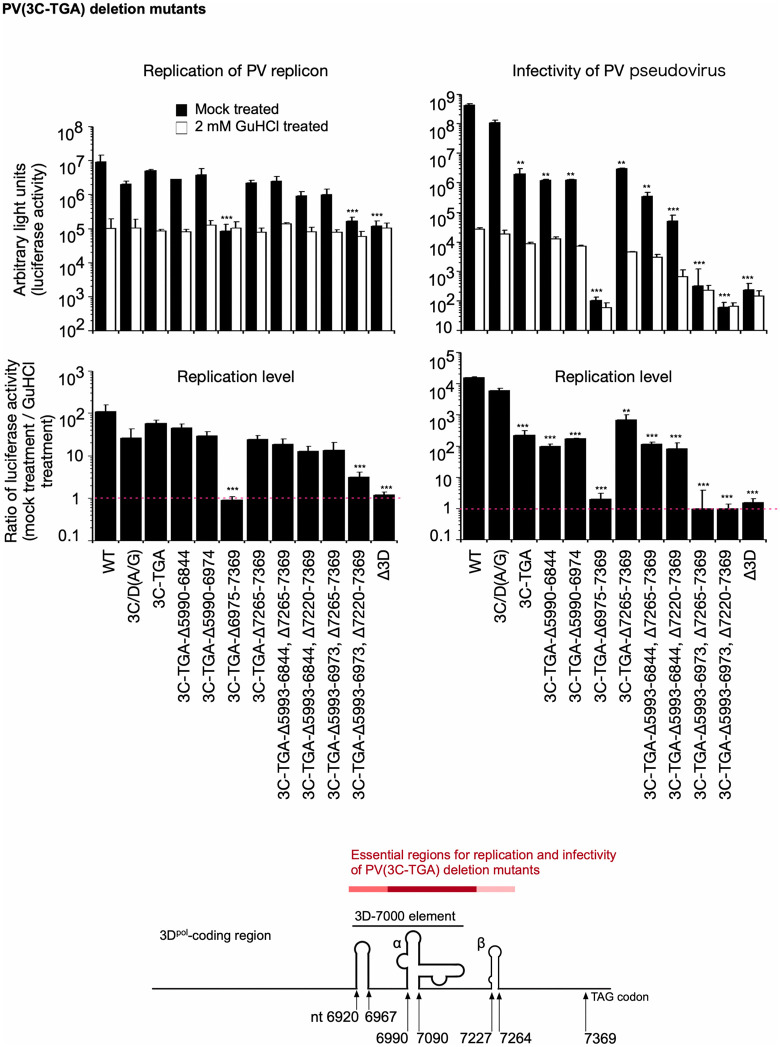
Replication and infectivity of PV mutants with deletions in the 3D^pol^-coding region. The luciferase signals measured at 7 h p.t. of RNA or 17 h p.i. with PV_pv_ are shown. Replication levels are the ratio of the luciferase activities observed in the absence of GuHCl to those observed in the presence of GuHCl. Identified essential genomic regions are highlighted in a schematic view of the 3D^pol^-coding region. The data represent the mean and standard deviation of three independent experiments with two biological replicates.

PV mutants with a termination codon immediately after the 3C^pro^-coding region, accompanied by deletions in the 3D^pol^-coding region showed that nt 6975–7369, nt-5993–6973 and nt 7265–7369 (positions of the nucleotides are of PV1(Mahoney) genome [GenBank accession number V01149.1]) are essential for the replication (**Fig 3**), consistent with previous reports [58,59]. The introduction of the termination codon immediately after the 3C^pro^-coding region (3C-TGA mutant) severely reduced the infectivity and replication level of PV_pv_ (approximately 1/100 of those of the parental 3C/D[A/G] mutant). However, replication levels of mutants measured by RNA transfection showed almost the same or slightly reduced levels as those of the parental 3C/D(A/G) mutant, except for those of lethal replicons (3C-TGA-Δ6975–7369 and Δ3D mutants). These results suggest that the 3CD^pro^ protein is essential for replication, as well as the RNA structures coded in the 3D^pol^-coding region.

**Fig 4:**
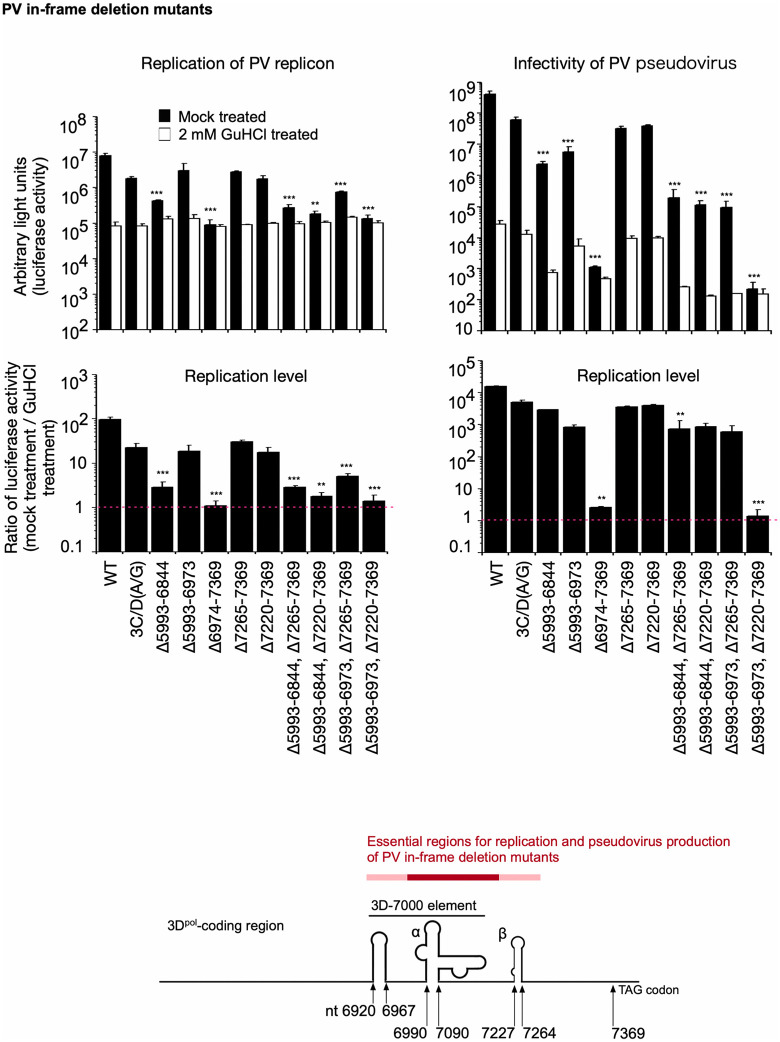
Replication and infectivity of PV mutants with in-frame deletions in the 3D^pol^-coding region. The luciferase signals measured at 7 h p.t. of RNA or 17 h p.i. with PV_pv_ are shown. Replication levels are the ratio of the luciferase activities observed in the absence of GuHCl to those observed in the presence of GuHCl. Identified essential genomic regions are highlighted in a schematic view of the 3D^pol^-coding region. The data represent the mean and standard deviation of three independent experiments with two biological replicates.

To identify the 3D^pol^ peptide region required in *cis* for *trans*-rescued replication, PV mutants that have in-frame deletions in the 3D^pol^ region were analyzed (**Fig 4**). The in-frame deletion immediately after the 3C^pro^-coding region severely affected infectivity, similar to that observed for those with a termination codon immediately after the 3C^pro^-coding region, and made a marked contrast to in-frame deletions of nt 7265–7369 or nt 7220–7369. Replication levels of the mutants, measured by transfection of RNA transcripts or PV_pv_ infection, showed a different profile, except for the Δ7265–7369 and Δ7220–7369 mutants, for which the replication level was comparable to that of the parental 3C/D(A/G) mutant. However, most of the mutants showed reduced infectivity in the presence of GuHCl, suggesting a defect in virion production. These results suggest that the 3D^pol^ peptides encoded immediately after the 3C^pro^-coding region or the coding region have essential roles in replication and virion production.

**Fig 5:**
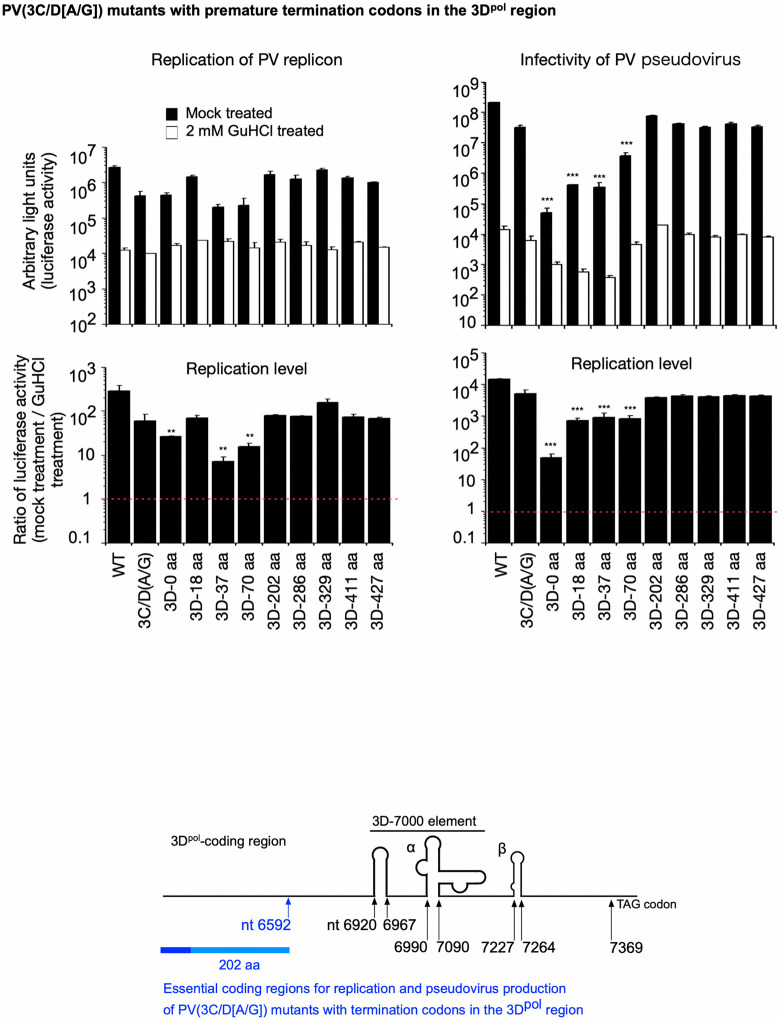
Replication and infectivity of PV mutants with termination codons (TGATAA) in the 3D^pol^-coding region. The luciferase signals measured at 7 h p.t. of RNA or 17 h p.i. with PV_pv_ are shown. Replication levels are the ratio of the luciferase activities observed in the absence of GuHCl to those observed in the presence of GuHCl. Identified essential coding regions are highlighted in a schematic view of the 3D^pol^-coding region. The data represent the mean and standard deviation of three independent experiments with two biological replicates.

To determine the length of the N-terminal peptide of 3D^pol^ required for efficient replication, PV mutants with premature termination codons in the 3D^pol^-coding region, thereby preserving the essential RNA structures, were analyzed (**Fig 5**). PV mutants that have less than 202 aa of the N-terminal 3D^pol^ showed reduced infectivity. Replication levels measured by RNA transfection or PV_pv_ infection showed a similar profile, except for the 3D-0 aa and 3D-18 aa mutants. These results suggest that at least the N-terminal 202 aa of 3D^pol^ are sufficient for efficient replication and virion production.

### Viral and host factors required for cleavage of 3AB *in vivo*

To analyze the effects of viral and host factors required for cleavage of 3AB *in vivo*, a polyprotein of PV non-structural proteins (2BC3ABCD, here after 2B2CP3) as a form of an N-terminally Azami green (AG)-fused protein (AG-2B2CP3), which allowed a high expression level of protein [26,62], with the indicated aa substitutions in the 3C^pro^ region or the premature termination codons in the 3D^pol^-coding region was expressed (**Fig 1A and 1D**). In this system, AG-2B2CP3 could be processed by the encoded 3C^pro^/3CD^pro^ in *cis* and *trans* in principle, depending on host factors, including PI4KB/OSBP and membrane structures in the cells. The effects of host factors PI4KB and OSBP on the cleavage in the polyprotein were analyzed using specific inhibitors to these proteins: T-00127-HEV1 (a PI4KB inhibitor) [49] and T-00127-HEV2, -HEV3, and -HEV4 (OSBP inhibitors) [35,62], which target OSBP with a high specificity among the members in the OSBP family [63]. To confirm the protease activity of the 3C^pro^/3CD^pro^, polyprotein processing was monitored using an anti-2C antibody.

**Fig 6:**
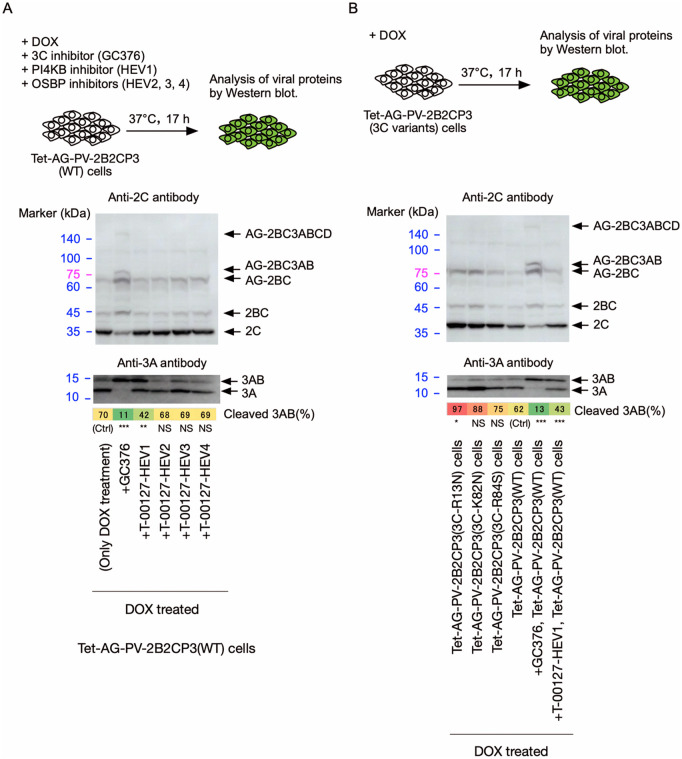
Effect of host factors and mutations on cleavage of 3AB *in vivo.* Western blot analysis of viral proteins in the cells expressing AG-PV-2B2CP3 proteins with the indicated amino acid substitutions or without the substitutions (*i.e.*, wild type, WT). The cells were treated with DOX (1 mg/L) for 17 h in the presence or absence of a 3C protease inhibitor (GC376, 100 μM), a PI4KB inhibitor (T-00127-HEV1, 10 μM), or OSBP inhibitors (T-00127-HEV2, -HEV3, and -HEV4, 10 μM). Viral proteins were detected by anti-3A or -2C antibodies. The percentage of cleaved 3AB is highlighted in color. The data are representative of three independent experiments with two to three biological replicates. NS, not significant.

Processing of the AG-PV-2B2CP3(WT) protein occurred efficiently, including that of 3AB (approximately 70% of 3AB was cleaved), which was almost completely blocked in the presence of GC376 (a 3C^pro^ inhibitor) as reported previously [26] (**Fig 6A**). Cleavage of 3AB was inhibited by a PI4KB inhibitor (T-00127-HEV1), albeit to a lesser extent than with GC376 treatment. Unexpectedly, the 3AB cleavage was not affected by OSBP inhibitors (T-00127-HEV2, -HEV3, and -HEV4). This result differs from that observed with an OSBP inhibitor, OSW-1, on processing of the polyprotein of coxsackievirus B3 (CVB3) [37]. Therefore, we attempted to evaluate the stability of the OSBP inhibitors used in this study *in vivo* (S1 Fig). Treatment of cells with OSBP inhibitors rapidly changes the cellular localization of OSBP from the cytosol to the Golgi [64], due to the accumulation of PI4P at the Golgi following the inhibition of PI4P/cholesterol transfer by OSBP, which is recognized by a pleckstrin homology domain of OSBP for its localization [43,65]. We used this phenomenon as a sensitive index of the intracellular activity of OSBP inhibitors. The intracellular activity of OSBP inhibitors was evaluated in HEK293 cells stably expressing C-terminally EGFP-fused OSBP (OSBP-EGFP), which is expressed approximately 10 times more than endogenous OSBP [62]. In untreated cells, OSBP-EGFP was mainly localized in the cytosol, but treatment with OSBP inhibitors caused the rapid relocalization to the Golgi. Treatment with T-00127-HEV4 caused the relocalization within 30 min, but the effect was diminished at 18 h. In contrast, in the cells treated with T-00127-HEV2- or -HEV3, the relocalization was maintained even after 18 h of treatment, consistent with the stability of these compounds in aqueous solution [63]. These results suggest that the inhibitory effects of T-00127-HEV2 and -HEV3 on endogenous OSBP in the cells can be maintained for up to 17 h after treatment. Therefore, the observed difference in the effects of OSBP inhibitors on 3AB cleavage seemed to reflect the intrinsic nature of the viral species (*Enterovirus betacoxsackie* or *Enterovirus coxsackiepol*), the action of OSBP inhibitors (*e.g.*, OSW-1 has specific activity to enhance degradation of OSBP, unlike other OSBP inhibitors) [66], or differences in the expression system of viral polyprotein.

AG-PV-2B2CP3 proteins with aa substitutions in the 3C^pro^ region, which could affect interaction with negatively charged molecules (3C-R13N, 3C-K82N, and 3C-R84S aa substitutions) [23,55–57], showed no defect in the processing of the polyprotein, including 3AB cleavage (**Fig 6B**), in contrast to the defects in replication or PV_pv_ infection caused by the individual aa substitution (**Fig 2B**). This suggests that PI4P produced by PI4KB is essential for the 3AB cleavage, but not via recruiting OSBP or 3C^pro^/3CD^pro^.

**Fig 7:**
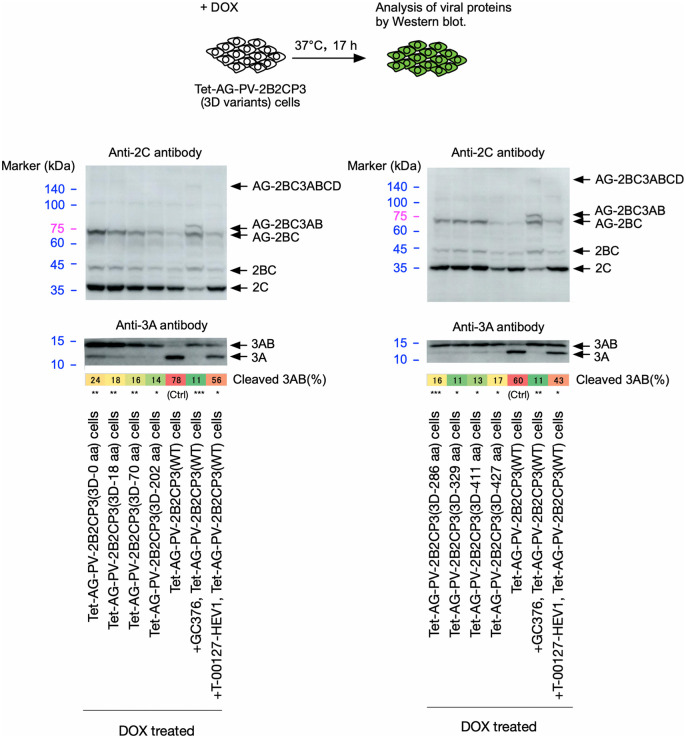
Effect of insertion of premature termination codons (TGATAA) in the 3D^pol^-coding region on cleavage of 3AB *in vivo.* Western blot analysis of viral proteins in the cells expressing AG-PV-2B2CP3 proteins with the indicated length of the 3D^pol^ peptides or without the insertion (*i.e.,* WT). The cells were treated with DOX (1 mg/L) for 17 h. The PV-2B2CP3(WT)-expressing cells were treated with DOX in the presence or absence of a 3C protease inhibitor (GC376, 100 μM) or a PI4KB inhibitor (T-00127-HEV1, 10 μM). Viral proteins were detected by anti-3A or -2C antibodies. The percentage of cleaved 3AB is highlighted in color. The data are representative of two independent experiments with two to three biological replicates. NS, not significant.

Introduction of premature termination codons in the 3D^pol^-coding region that result in the addition of the N-terminal 0, 18, 70, 202, 286, 329, 411, and 427 aa of 3D^pol^ to 3C^pro^ suppressed the 3AB cleavage (approximately only 20% of 3AB was cleaved), without affecting other processing of the polyprotein (**Fig 7**). This suggests that almost the entire region of 3D^pol^ is required for cleavage of 3AB in polyprotein *in vivo*.

### Genome structure of PV_pv_ pseudorevertants

Efficient cleavage of 3AB requires almost the entire region of the 3D^pol^-coding region (**Fig 7**), posing a contradiction with the observed replication of PV mutants (**Fig 5**). To analyze the potential role of revertants in the observed replication, the stability of the introduced mutations in the genomes of PV_pv_ mutants, which were obtained after two rounds of passage, was analyzed. After two rounds of passage, most of the mutants exhibited a drastic increase in infectivity and replication level (approximately 10–1000-fold) (S2 Fig), suggesting that the mutants are quasi-infectious. A maximum of two independent clones per examined mutant were isolated after passage (a total of 46 clones) and subjected to whole-genome sequencing analysis by nanopore sequencing. The genomes of most mutants exhibited reversion or pseudoreversion (S1 Data); however, unexpectedly, this was not observed in the Δ7265–7369 and Δ7220–7369 mutants, as well as PV mutants that had 202 aa or more of the N-terminus of 3D^pol^ (**Table 1**). The genomes of the 3C-TGA mutants exhibited pseudoreversions, changing the introduced termination codon (TGA) to codons for cysteine (TGT or TGC) or tryptophan (TGG), thus regaining the following 3D^pol^ peptides. The genomes of PV mutants with premature termination codons in the 3D^pol^-coding region and less than 202 aa of the N-terminal 3D^pol^ exhibited in-frame deletion or duplication to remove the introduced termination codon (TGATAA) and acquired an increased length of peptides after the 3C^pro^ (186–437 aa), supporting that more than 70 aa of the N-terminal 3D^pol^ after 3C^pro^ is required for efficient replication. Interestingly, the genomes of PV with deletions in the 3D^pol^-coding region showed extensive in-frame duplication (**Table 1**). The in-frame duplication showed the junctions of 3C/2C, 3C/3B, 3C/3C, 3D/2C, and 3D/3C. The genomes of some pseudorevertants (*i.e.*, 3C-TGA-Δ5993–6844 revertant clone 1, 3C-TGA-Δ5993–6974 revertant clone 1, 3C-TGA-Δ5993–6844, Δ7220–7369 revertant clone 1) showed the junction of 3C/2C, thus had almost two sets of the P3-coding region (*i.e.*, the genome structure of 2B2C3A3B3C[partial]/2C[partial]3A3B3C3D[partial]) (S3 Fig). The in-frame duplication generally resulted in increased length of peptides after 3C^pro^, but not for some pseudorevertants (Δ5993–6844 revertant clone 1, Δ5993–6973 revertant clone 1, and Δ5993–6844, Δ7220–7369 revertant clone 1), suggesting that the length of the peptide after 3C^pro^ is not the sole determinant that caused the duplication for these deletion mutants. The total length of the genomes of these pseudorevertants was consistently increased by duplication (an increment of 332–2139 nt), suggesting a minimum genome length of 5800 nt (without poly A) may be required to support efficient replication and virion production of these deletion mutants. To analyze the effect of genome length on in-frame duplication, the genomes of PV_pv_ with a mCherry reporter (WT and 3C/D[A/G] mutant) (a genome length of 5580 nt and without poly A) [67] obtained after two rounds of passage were analyzed. No mutation was found in the genomes of the WT and 3C/D(A/G) mutant with a mCherry reporter (**Table 1**), suggesting that deletion in the 3D^pol^-coding region is the determinant for the in-frame duplication rather than the genome length. In summary, these results suggest that viral genomes with deletions in the 3D^pol^-coding region are genetically unstable and regain infectivity via extensive in-frame duplication.

**Table 1:** Genome structures of PV_pv_ clones obtained after two rounds of passage.

PV pseudovirus mutants	Serial number of isolated clones	Mutations in the viral genome^*a*^	Junctions of recombination	Length of insertion (nt)	Length of peptides after 3C in the isolates (aa)	Length of peptides after 3C in the original constructs (aa)	Estimated length of the genomes of isolates (nt)	Length of the genomes of original clones (nt)	Change of the length of the genomes (isolates - original) (nt)
WT	1	No mutation	NA	NA	461	461	6477	6477	0
	2	No mutation	NA	NA	461	461	6477	6477	0
3C/D(A/G)	1	No mutation	NA	NA	461	461	6477	6477	0
	2	No mutation	NA	NA	461	461	6477	6477	0
3C-TGA	1	Substitution (3C-TGA to TGT(Cys))	NA	NA	461	0	6477	6477	0
	2	Substitution (3C-TGA to TGG(Trp))	NA	NA	461	0	6477	6477	0
3C-TGA-Δ5990–6844	1	In-frame duplication (nt 5977/4676)	3C/2C	1302	145	0	6922	5622	1300
	2	In-frame duplication (nt 5981/5553)	3C/3C	429	145	0	6050	5622	428
3C-TGA-Δ5990–6974	1	In-frame duplication (nt 5986/4619)	3C/2C	1368	164	0	6873	5492	1381
3C-TGA-Δ6975–7369	No pseudovirus isolated	NA	NA	NA	NA	0		6082	
3C-TGA-Δ7265–7369	1	Substitution (3C-TGA to TGC(Cys)), A7054G (silent mutation)	NA	NA	426	0	6372	6372	0
3C-TGA-Δ5990–6844, Δ7265–7369	1	In-frame duplication (nt 5978/5382)	3C/3B	597	19	0	6113	5517	596
3C-TGA-Δ5990–6844, Δ7220–7369	1	In-frame duplication (nt 5987/5457)	3C/3C	531	177	0	6002	5472	530
	2	In-frame duplication (nt 5973/4273)	3C/2C	1701	277	0	7171	5472	1699
3C-TGA-Δ5990–6973, Δ7265–7369	No pseudovirus isolated	NA	NA	NA	NA	0		5391	
3C-TGA-Δ5990–6973, Δ7220–7369	No pseudovirus isolated	NA	NA	NA	NA	0		5346	
Δ5993–6844	1	In-frame duplication (nt 6875/5496)	3D/3C	1380	176	177	6152	5625	527
	2	In-frame duplication (nt 6900/5467)	3D/3C	1434	194	177	6206	5625	581
Δ5993–6973	1	In-frame duplication (nt 6996/5710)	3D/3C	1287	102	134	5800	5497	303
	2	In-frame duplication (nt 7098/5668)	3D/3C	1431	150	134	5945	5497	448
Δ6974–7369	No pseudovirus isolated	NA	NA	NA	NA	329		6081	
Δ7265–7369	1	C45T (5’NTR)	NA	NA	426	426	6372	6372	0
	2	T6973C (silent mutation)	NA	NA	426	426	6372	6372	0
Δ7220–7369	1	No mutation	NA	NA	411	411	6327	6327	0
	2	No mutation	NA	NA	411	411	6327	6327	0
Δ5993–6844, Δ7265–7369	1	In-frame duplication (nt 6874/5450)	3D/3C	1425	199	142	6116	5520	596
	2	In-frame duplication (nt 6855/5407)	3D/3C	1449	191	142	6092	5520	572
Δ5993–6844, Δ7220–7369	1	In-frame duplication (nt 6891/5707)	3D/3C	1185	111	127	5807	5475	332
Δ5993–6973, Δ7265–7369	1	In-frame duplication (nt 7245/4819)	3D/2C	(2427)	>147	99	7530	5391	2139
Δ5993–6973, Δ7220–7369	1	In-frame duplication of (nt 7041/5449)	3D/3C	1593	204	84	5957	5346	611
3D-0 aa	1	In-frame deletion (nt 5986–6455), T566C (5’NTR)	3C/3D	NA	305	0	6008	6477	-469
3D-18 aa	1	In-frame deletion (nt 6022/6410), G4850A(2C-A243T), A5239G(3A-I43M)	3D/3D	NA	332	18	6089	6477	-388
	2	In-frame duplicatioin nt 6019/5516)	3D/3C	504	186	18	6981	6477	504
3D-37 aa	1	In-frame deletion (nt 6021/6436), C739A (5’NTR)	3D/3D	NA	323	37	6062	6477	-415
3D-70 aa	1	In-frame deletion (nt 6181/6254)	3D/3D	NA	437	70	6404	6477	-73
	2	In-frame deletion (nt 6189/6262)	3D/3D	NA	437	70	6404	6477	-73
3D-202 aa	1	A5116G (silent mutation)	NA	NA	202	202	6477	6477	0
	2	No mutation	NA	NA	202	202	6477	6477	0
3D-286 aa	1	No mutation	NA	NA	286	286	6477	6477	0
	2	No mutation	NA	NA	286	286	6477	6477	0
3D-329 aa	1	No mutation	NA	NA	329	329	6477	6477	0
	2	No mutation	NA	NA	329	329	6477	6477	0
3D-411 aa	1	No mutation	NA	NA	411	411	6477	6477	0
	2	No mutation	NA	NA	411	411	6477	6477	0
3D-427 aa	1	A4386G(2C-K88R)	NA	NA	427	427	6477	6477	0
	2	No mutation	NA	NA	427	427	6477	6477	0
WT (mCherry reporter)	1	No mutation	NA	NA	461	461	5580	5580	0
	2	No mutation	NA	NA	461	461	5580	5580	0
3C/D(A/G) (mCherry reporter)	1	No mutation	NA	NA	461	461	5580	5580	0
	2	No mutation	NA	NA	461	461	5580	5580	0

^*a*^ The positions of the nucleotides are of PV1(Mahoney) genome (GenBank accession number V01149.1). Length of insertion inferred from the obtained partial nt sequences is shown in parentheses.

### 3CD^pro^ or 3D^pol^ provided in *trans* can rescue a defect in the cleavage of 3AB

**Fig 8:**
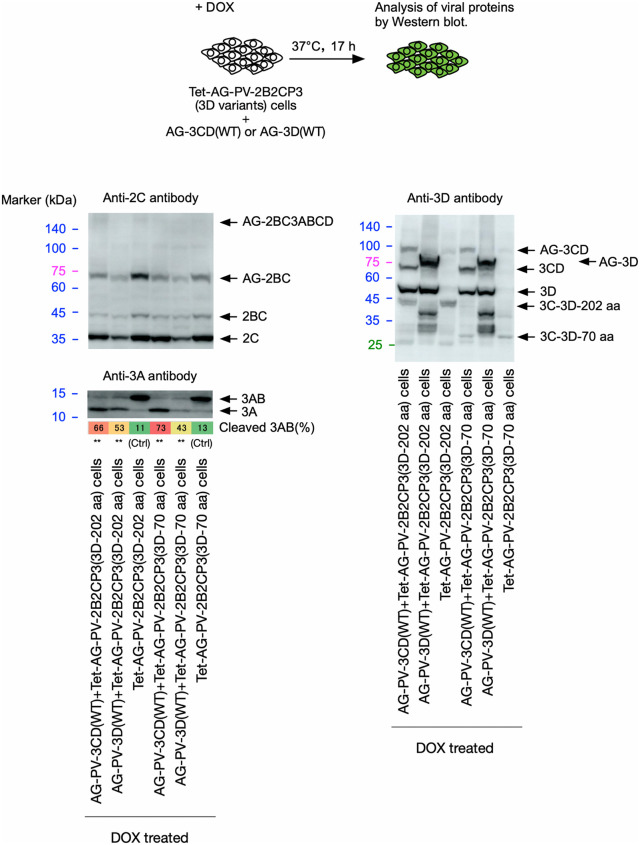
The effect of 3CD^pro^ and 3D^pol^ provided in *trans* on the cleavage of 3AB *in vivo.* Western blot analysis of viral proteins in the cells expressing AG-PV-2B2CP3 proteins that have premature termination codons (TGATAA) in the 3D^pol^-coding region, resulting in partial 3D^pol^ peptides as indicated (70 or 202 aa of the N-terminal peptides), along with AG-3CD(WT) or AG-3D(WT) proteins. The cells were treated with DOX (1 mg/L) for 17 h. Viral proteins were detected by anti-3A, -2C, or -3D antibodies. The percentage of cleaved 3AB is highlighted in color. The data are representative of three independent experiments with two biological replicates. NS, not significant.

PV mutants that encode equal or more than 202 aa of the N-terminal 3D^pol^, which should have a defect in cleavage of 3AB (**Fig 7**), replicated efficiently and stably without reversion/pseudoreversion (S2 Fig **and Table 1**), posing an apparent contradiction. This prompted us to analyze the role of 3CD^pro^ provided in *trans* in the cleavage of 3AB. 3CD^pro^ or 3D^pol^, as a form of an N-terminally AG-fused protein, was co-expressed with the polyproteins that have the premature termination codons in the 3D^pol^-coding region, resulting in the addition of the N-terminal 70 or 202 aa of 3D^pol^ (**Fig 8**). While the PV mutant 3D-202 aa showed stable *trans*-rescued replication, the PV mutant 3D-70 aa was quasi-infectious, indicating a defect in the *trans*-rescued replication (S2 Fig **and Table 1**). Co-expression of AG-3CD or AG-3D slightly affected the expression of the polyproteins, probably due to their high-level expression that could cause competition in transcription/translation, in contrast to AG-3C expression (S4 Fig), but not the processing of 2B2C. The defect of the polyproteins (AG-2B2CP3[3D-202 aa] and AG-2B2CP3[3D-70 aa]) in the 3AB cleavage was rescued by the co-expression of AG-3CD or AG-3D, indicating that the 3D^pol^ region provided in *trans* is essential to rescue the defect in the cleavage of 3AB. These results suggest that the 3D^pol^ region provided in *trans* can rescue the defect in the 3AB cleavage of polyproteins or PV mutants, irrespective of the length of the encoded 3D^pol^ peptides.

**Fig 9:**
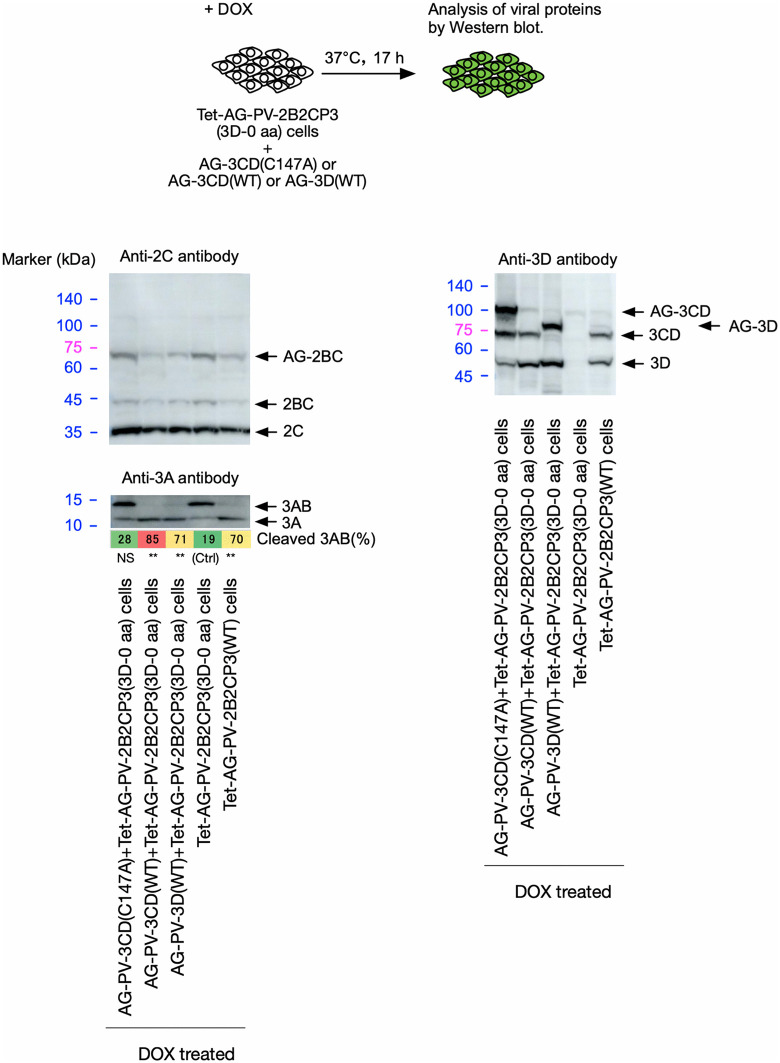
Effect of 3CD^pro^ activity provided in *trans* on cleavage of 3AB *in vivo.* Western blot analysis of viral proteins in the cells co-expressing AG-PV-2B2CP3(3D-0 aa) and AG-3CD variants (WT or C147A) or AG-3D(WT). The cells were treated with DOX (1 mg/L) for 17 h. Viral proteins were detected by anti-2C, -3A, or -3D antibodies. The percentage of cleaved 3AB is highlighted in color. The data are representative of three independent experiments with two to three biological replicates. NS, not significant.

The 3AB cleavage in a polyprotein encoding an inactive 3C^pro^/3CD^pro^ was not rescued by an active 3 CD^pro^ provided in *trans* [26]. To further determine the mode of action of 3C^pro^/3CD^pro^ in 3AB cleavage in a polyprotein encoding an active 3C^pro^, the potential *trans* role of 3CD^pro^ in the cleavage was evaluated (**Fig 9**). An inactive 3CD^pro^ variant (C147A) was co-expressed with an AG-2B2CP3(3D-0 aa) polyprotein variant, which completely lacks 3D^pol^ peptides. The expressed AG-3CD(C147A) is processed only by 3C^pro^ encoded in the polyprotein in *trans*, showing different processing profiles compared to AG-3CD(WT), with a large amount of the precursor AG-3CD(C147A) remaining intact. Nevertheless, a substantial amount of 3CD^pro^(C147A), similar to that of 3CD^pro^(WT) produced from AG-3CD(WT), and a slightly smaller amount of 3D^pol^(WT) were produced from AG-3 CD(C147A). Interestingly, the co-expression of AG-3CD(C147A) did not facilitate 3AB cleavage in the AG-2B2CP3(3D-0 aa) polyprotein, in marked contrast to the co-expression of AG-3CD(WT) or AG-3D(WT). This suggests that AG-3CD(C147A) or 3CD^pro^(C147A) antagonized the *trans* effect of 3D^pol^(WT) produced from AG-3CD(C147A) to facilitate 3AB cleavage. To evaluate the potential dominant negative effect of AG-3CD(C147A) or 3CD^pro^(C147A) on 3AB cleavage, AG-3CD(C147A), AG-3CD(WT), or AG-3D(WT) was co-expressed with an AG-2B2CP3(WT) polyprotein, in which 3AB is cleaved by 3C^pro^/3 CD^pro^ encoded in the polyprotein (S5 Fig). The 3AB cleavage was not affected by the co-expression with AG-3CD(WT) or AG-3D(WT), but was partially suppressed by the co-expression with AG-3CD(C147A). This suggested that 3C^pro^/3CD^pro^ could affect 3AB cleavage in *trans*. Taken together, these results suggest that 3AB cleavage requires protease activity in *cis*, but the efficiency of cleavage is strongly affected by *trans*-acting viral/host proteins and cellular context, suggesting a hybrid mechanism.

**Fig 10:**
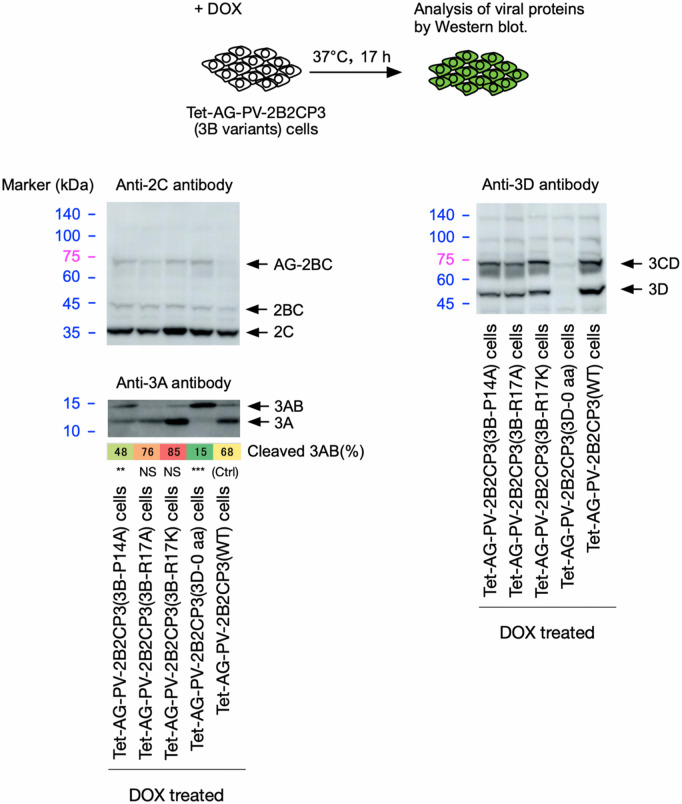
Effect of aa substitution in the 3B region of a PV polyprotein on the cleavage of 3AB *in vivo.* Western blot analysis of viral proteins in the cells expressing AG-PV-2B2CP3 variants. The cells were treated with DOX (1 mg/L) for 17 h. Viral proteins were detected by anti-2C, -3A, or -3D antibodies. The percentage of cleaved 3AB is highlighted in color. The data are representative of three independent experiments with two biological replicates. NS, not significant.

**Fig 11:**
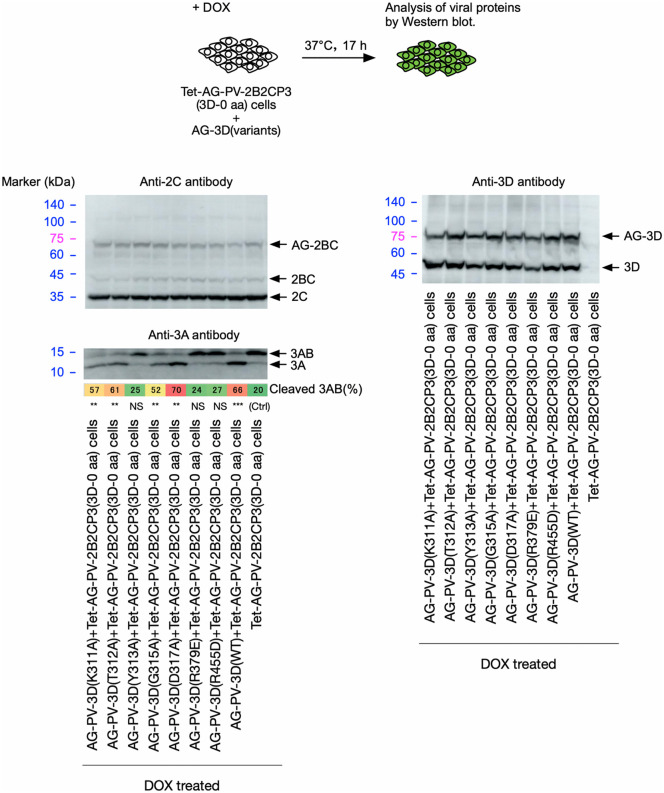
Effect of 3D^pol^ variants provided in *trans* on cleavage of 3AB *in vivo.* Western blot analysis of viral proteins in the cells co-expressing AG-PV-2B2CP3(3D-0 aa) and AG-3D variants. The cells were treated with DOX (1 mg/L) for 17 h. Viral proteins were detected by anti-2C, -3A, or -3D antibodies. The percentage of cleaved 3AB is highlighted in color. The data are representative of two independent experiments with two biological replicates. NS, not significant.

To assess the importance of the specific functional interaction of 3D^pol^ provided in *trans* with 3AB in the cleavage, rather than the potential indirect effect via modification of the cellular environment, we analyzed the effect of aa substitution of the following aa residues in 3B and 3D^pol^ involved in 3AB-3D^pol^ or 3D^pol^ -3D^pol^ interactions, on the cleavage (**Figs 10 and 11**): P14 and R17 in 3B, which are essential for the binding of 3AB to 3D^pol^ [68], R379 in 3D^pol^, which is essential for the binding of 3AB to 3D^pol^ and uridylation of 3B [69,70], K311, T312, Y313, G315, D317 in 3D^pol^ and P14 and R17 in 3B, which are located near the binding sites of 3D^pol^ and 3AB on the structural models predicted by Alphahold3 (S6 Fig), R455 in 3D^pol^, which is essential for the interaction between 3D^pol^ and 3D^pol^ at the interface I [71] and also for the uridylation of 3B [72]. The aa substitutions in 3B were introduced in a polyprotein AG-2B2CP3, and those in 3D^pol^ were introduced in AG-3D, which was co-expressed with a polyprotein AG-2B2CP3(3D-0 aa) that completely lacked 3D^pol^ peptides.

The P14A substitution in a polyprotein significantly suppressed cleavage of 3AB, whereas the R17A and R17K substitutions showed no effect (**Fig 10**). While the expression of AG-3D variants (K311A, T312A, and D317A) facilitated the cleavage of 3AB in AG-2B2CP3 (3D-0 aa) as well as AG-3D(WT), the expression of AG-3D variants (Y313A, R379E, and R455D) completely lost the *trans* activity to support the 3AB cleavage. Expression of an AG-3D variant (G315A) only partially facilitated the cleavage, in contrast to AG-3D(WT) (**Fig 11**). These results suggest that cleavage of 3AB requires 3D^pol^-3AB and 3D^pol^-3D^pol^ interactions via P14 in 3B and Y313, G315, R379, and R455 in 3D^pol^.

These results suggest that the 3D^pol^ region provided in *trans* can rescue the defect in 3AB cleavage of the PV polyprotein, independently of the 3D^pol^ peptides encoded in the polyprotein. The observed defect in replication of the PV mutant 3D-70 aa may occur at an uncharacterized step in addition to the 3AB cleavage.

## Discussions

In this study, we investigated the role of 3CD^pro^ in the cleavage of PV 3AB *in vivo* (*i.e.*, in cultured cells). 3CD^pro^ is a multifunctional RNA-binding protease that plays key roles in the replication, including efficient cleavage of P1 [3,22], switching of the viral genome from translation to RNA replication [23,24], stimulation of uridylylation of 3B [25], *cis* role in the cleavage of 3AB and provision of 3D^pol^ activity in *trans* [26]; however, the mechanism that supports these function remains largely unknown. As a mechanism of the 3CD^pro^ function, the binding activity of the 3C^pro^ region to negatively charged molecules (RNA and phospholipids) has been reported [23,55–57]. Two 3C^pro^ molecules could bind to an RNA stem-loop in the 5’ cloverleaf structure of EV genomes [73]. PV mutants that lack this activity are lethal or quasi-infectious [61], underscoring the importance of this activity in infection. PV mutants with 3C-R13N, 3C-K82N, or 3C-R84S aa substitutions, which affect the binding activity [23,55–57], showed severely suppressed *trans*-rescued replication (**Fig 2B**), suggesting a critical *cis* role of binding activity of 3C^pro^/3CD^pro^ to negatively charged molecules.

In PV replication, host PI4KB provides PI4P, a negatively charged lipid, on the viral RO [16]. Therefore, the binding of 3CD^pro^ to PI4P was considered a candidate mechanism to support the cleavage of 3AB. However, polyproteins that encode the 3CD^pro^ variants showed efficient cleavage of 3AB (**Fig 6B**). Nevertheless, the activity of PI4KB was required for the cleavage, in contrast to the inhibition of OSBP, which had little effect on the cleavage in a polyprotein (**Fig 6A**). These results suggest that PI4P is essential for cleavage of 3AB but plays a role distinct from the recruitment of 3CD^pro^ or OSBP to the RO for this step. PI4KB and OSBP support viral replication via the same functional pathway in EV replication [51]; however, PI4KB may have a more specific role in cleavage of 3AB in PV replication. In PV replication, resistance mutations in 3A, which promote cleavage of 3AB [36], showed a relatively weak effect against OSBP inhibitors compared to PI4KB inhibitors [50]. Observed 3AB cleavage in a polyprotein in the presence of OSBP inhibitors may suggest OSBP is involved in the function of 2B downstream of the 3AB cleavage in PV replication [50,54]. In support of this view, in encephalomyocarditis virus (EMCV) replication, a resistance mutation in 3A conferred resistance to a PI4KA (one of the four mammalian PI4Ks) inhibitor but not to OSBP inhibitors [74]. On the other hand, in CVB3 replication, a resistance mutation in 3A conferred a similar level of resistance to both a PI4KB inhibitor and to OSBP inhibitors [74,75]. These findings suggest that the roles of PI4KB/PI4KA and OSBP in 3AB cleavage vary among picornaviruses, and that OSBP may have a different function downstream of PI4KB/PI4KA activity.

The 3D^pol^ region of the PV genome has RNA structures that are important for replication and infectivity, named as α (nt 6995–7069 in PV1[Mahoney] genome), β (nt 7227–7264 in PV1[Mahoney] genome) [58], and 3D-7000 (nt 6920–7090 in PV1[Mahoney] genome) [59]. These structures are conserved in *Enterovirus coxsackiepol* species and required for replication and infectivity [58,59]. Since replication of a PV mutant that lacks the entire 3D^pol^-coding region could not be *trans*-rescued by 3CD^pro^, these RNA structures should function in *cis* [26]. Consistent with a previous report [58], PV mutants with a deletion of both α and β were lethal (**Figs 3**–**5**). Only the 3D^pol^ region that is dispensable in *trans*-rescued replication was located between nt 7220 and 7369; PV mutants that had a deletion just before or within 3D-7000 were quasi-infectious and caused pseudoreversions after passages. Interestingly, all of these pseudorevertants exhibited extensive in-frame duplications (**Table 1 and** S3 Fig), some of which contained nearly two sets of the P3 region in their genomes, including 3B. Some uncharacterized activity of the large polyprotein precursor, P3, might have been required to support the replication of these mutants [60,76]. PV mutants with premature termination codons in the 3D^pol^-coding region showed in-frame deletion rather than in-frame duplication after passages (**Table 1**). This suggested that deletion of the 3D^pol^-coding region, rather than the 3D^pol^ peptide, was the driving force behind the in-frame duplication. In-frame duplication or non-homologous recombination between non-replicable EV RNA and replicable EV RNA has been well established [77,78]. The genome duplication observed in the current study was significantly larger (429–1593 nt) than that observed in non-replicable EV RNA recombination (approximately 100 nt), suggesting a functional requirement of the genome duplication to complement the uncharacterized defects in replication of the PV mutants. Only two genera in the family *Picornaviridae* (comprising 63 genera), *i.e., Aphthovirus* and *Mosavirus*, have multiple copies of 3B (three and two copies, respectively) in the genomes; however, two copies of 3B are unstable in the PV genome [79,80]. Therefore, acquiring multiple copies of 3B in picornavirus genomes may be a rare event in picornavirus evolution. A gene produced by duplication could evolve to have different functions [81]; the 3B1 gene in the FMDV genome is one such example in picornavirus evolution [82]. Gene duplication does not occur frequently in the evolution of RNA viruses [83]; some special interactions between picornaviruses, including *trans* complementation, might have triggered such a rare event during evolution.

The 3AB plays multiple roles in replication, in addition to providing 3B, which could serve as the primer for viral RNA synthesis after uridylylation [11], including stimulation of 3D^pol^ polymerase activity [19,20] and processing of 3CD^pro^ [84]. The pathway to produce the functional uridylylated 3B as the primer for viral RNA synthesis has yet to be determined, since the 3B region of 3BC and 3BCD can also serve as substrates for uridylylation *in vitro* and can be found covalently linked to viral RNA *in vivo* [85,86], as well as 3B itself. On the other hand, *cis*-complementation studies have suggested that 3AB is the most likely precursor of 3B used for uridylylation *in vivo* [87]. Efficient *trans*-rescued replication of PV mutants that exclusively produce 3CD^pro^ but not 3C^pro^, suggesting that 3CD^pro^ could play the *cis* role of 3C^pro^, including the 3AB cleavage [26]. Cleavage of 3AB required the entire 3D^pol^ region of 3CD^pro^ encoded in the polyprotein (**Fig 7**). Surprisingly, PV mutants that could express the N-terminal 202 aa or more of 3D^pol^ peptides can stably maintain the introduced mutations in *trans*-rescued replication (**Table 1**). We found that 3D^pol^ provided in *trans* could rescue the defect in 3AB cleavage in the corresponding polyproteins (**Figs 8 and 9**). The effect of 3D^pol^ or 3CD^pro^ provided in *trans* on the 3AB cleavage was expression level-dependent and saturable (S7 Fig). Therefore, the 3D^pol^ region seems to function in *trans* in the 3AB cleavage, and in *cis* in the replication with the RNA structures coded in this region [58,59] and in the genetic stability of viral genome with the 3D^pol^ peptides (**Table 1**), via different regions (**Figs 3**–**5, and 7**). A *cis* role of 3D^pol^ in foot-and-mouth disease virus (FMDV) replication is involved in the replication via interaction with the 5’ untranslated region of the viral genome rather than via polyprotein processing [88]. To gain insight into the observed effect of the 3D^pol^ region, structural models were predicted using AlphaFold3 (S6 Fig), which include one to two molecules of 3AB and 3D^pol^, considering both homotypic and heterotypic interactions between 3AB and 3D^pol^ [68,69,89–91]. It should be noted that the confidence metrics for the 3AB structures were generally low (pLDDT < 70) in the proposed models, likely reflecting the intrinsic disordered nature of 3AB. In addition, the models do not include contributions from other viral/host factors (e.g., proteins, RNA, and lipids) to recapitulate the 3D^pol^-3AB interaction within a replication complex *in vivo*. Despite these limitations, some features established by previous studies were reproduced in the models, including the involvement of the 3B region in the 3D^pol^-3AB interaction [68,90] and the interface I in the 3D^pol^-3D^pol^ interaction [71]. However, the predicted binding sites of 3B on PV 3D^pol^ in the models (around Y313) were far from those suggested in PV 3D^pol^ (around R379) [69,70,89] and in a crystal structure of CVB3 3D^pol^ (around Y378) [92], but relatively close to that suggested in EV-A71 3D^pol^ (around T313) [93]. The Y313 and G315 residues of PV 3D^pol^ are located near the interface I, suggesting a potential involvement in the 3D^pol^-3D^pol^ interaction. We found that aa substitutions at P14 in 3B and at Y313, G315, R379, and R455 in 3D^pol^ could significantly affect 3AB cleavage (**Figs 10 and 11**), indicating a remarkable overlap between the aa residues required for 3AB cleavage with those required for the uridylylation of 3B *in vitro*, including those required for the binding of 3B to 3D^pol^ as a prerequisite of the reaction [69,70,72,94]. This might suggest that cleavage of 3AB and the subsequent uridylylation of 3B occur in a coupled reaction to enhance the efficiency of uridylylation *in vivo.*

The limitations of this study include the unknown mechanism of in-frame genome duplication by which PV mutants with partial deletions in the 3D^pol^ region regain fitness during *trans*-rescued replication. Because the defect of the 3AB cleavage of polyproteins could be rescued by 3D^pol^ provided in *trans*, independently of the 3D^pol^ peptides encoded in the polyproteins (**Figs 8**–**10**), it is plausible that some targets other than the 3AB cleavage would also have been involved in the *trans*-rescued replication and in the genetic stability of the viral genome via the peptides following 3C^pro^. Elucidation of the binding site of PV 3B on 3D^pol^ will unambiguously clarify the role of aa residues examined in this study in 3AB cleavage, including Y313 of 3D^pol^.

Collectively, this study reveals novel roles for the 3D^pol^ region in cleavage of PV 3AB and in the genetic stability of the viral genome. Our findings might be useful for the development of effective antivirals targeting the polyprotein processing and viral genome recombination.

## Materials and methods

### Cells

RD cells (human rhabdomyosarcoma cells) and HEK293 cells (human embryonic kidney cells) were cultured as monolayers in Dulbecco’s modified Eagle medium (DMEM) supplemented with 10% foetal calf serum (FCS). RD cells were used for virus titration. HEK293 cells were used for expression of PV non-structural proteins and for production of PV pseudovirus (PV_pv_).

### Viruses

PV_pv_ was produced with a firefly luciferase-coding or a mCherry-coding type 1 PV (PV1) Mahoney strain (GenBank accession number V01149.1) replicon and capsid proteins of PV1(Mahoney) [67,95].

### Antibodies

Rabbit hyperimmune serum against PV 3A and 3D proteins were prepared in previous studies [36,51,96]. Rabbit hyperimmune serum against PV 2C was a kind gift from Tomoichiro Oka (National Institute of Health Sciences, Japan).

### Chemicals

Doxycycline (DOX) was purchased from FUJIFILM Wako Pure Chemical Corporation (049–31121). Guanidine hydrochloride (GuHCl, a 2C inhibitor) was purchased from SIGMA (G-9284). GC376 (a 3C inhibitor) was purchased from Selleck Chemicals (S0475). T-00127-HEV1 was purchased from Pharmeks Ltd., Moscow, Russia (purity >99%). T-00127-HEV2 was kindly provided by Hirotatsu Kojima (Open Innovation Center for Drug Discovery, University of Tokyo, Tokyo, Japan, purity >99%). T-00127-HEV3 and -HEV4 were purchased from Enamine (#Z49616154 [purity 92%] and #Z49616105 [purity 92%], respectively). Stock solutions of DOX (2 g/L) and GuHCl (2 M) were prepared in Milli-Q water. Stock solutions of GC376 (100 mM) and rupintrivir (10 mM) were prepared in dimethyl sulfoxide.

### General methods for molecular cloning

*Escherichia coli* strain XL10-Gold (Agilent, #200314) was used for the preparation of plasmids. Ligation of DNA fragments was performed using an NEBuilder HiFi DNA Assembly Master Mix (NEB, #E2621L). PCR was performed using KOD FX Neo DNA polymerase (Toyobo, #KFX-201X5). Sanger DNA sequencing was performed to confirm the presence of introduced mutations in the plasmids using a BigDye Terminator v3.1 cycle sequencing ready reaction kit (Applied Biosystems) and then analyzed with a 3500xL genetic analyzer (Applied Biosystems). Viral RNA was extracted from 200 μL of culture supernatant of PV_pv_-infected cells using a *Quick*-RNA Viral Kit (ZYMO RESEARCH, #R1035).

### Plasmids

#### Lentivirus expression vectors for PV capsid proteins.

pTet-AG-PV1(Mahoney) capsid:

A cDNA fragment of the coding region of the capsid proteins of PV1(Mahoney) strain was obtained by PCR with pKS435-EGFP-PV CAPSID [95] as the template and following primer set 1. This DNA fragment was inserted into a DNA fragment of a lentivirus vector plasmid with a TRE3G promoter, which was obtained by PCR with pLJM1-TRE3G-His-AG-FLAG-PreScission-OSBP(406–807) [62] as the template and using primer set 2.

Primer set 1:

5’ GAAGTTCTGTTCCAGGGCGCCCAGGTTTCATCACAGAAAGTGGGC

3’

5’ TCTGAGTCCGGATCAATATGTGGTCAGATCCTTGGTGGAGAGG

3’

Primer set 2:

5’ CTGGAACAGAACTTCCAGCTTGTCGTCATC 3’

5’ TGATCCGGACTCAGATCTCGAGCTCAAGC 3’

#### Lentivirus expression vectors for PV non-structural proteins.

pTet-AG-PV-2B2CP3(3C-R13N, 3C-K82N, 3C-R84S, 3B-P14A, 3B-R17A, 3B-R17K):

Mutations for the aa substitutions were introduced in pTet-AG-PV-2B2CP3(WT) by PCR with primer sets in S1 Table.

pTet-AG-PV-2B2CP3(3D-0 aa, 3D-18aa, 3D-37aa, 3D-70 aa, 3D-202 aa, 3D-286 aa, 3D-329 aa, 3D-411 aa, 3D-427 aa):

Tandem termination codons (TGATAA) after the indicated sites in the 3D^pol^ region were introduced in pTet-AG-PV-2B2CP3(WT) by PCR with primer sets in S1 Table.

pTet-AG-PV-3CD(C147A):

Mutations for the aa substitution were introduced in pTet-AG-PV-3 CD(WT) by PCR with primer sets in S1 Table.

pTet-AG-PV-3D(K311A, T312A, Y313A, G315A, D317A, R379E, and R455D):

Mutations for the aa substitutions were introduced in pTet-AG-PV-3D(WT) by PCR with primer sets in S1 Table.

### PV replicon mutants

pPV-Fluc mc (3C/D[A/G]-3C-R13N, 3C/D[A/G]-3C-K82N, 3C/D[A/G]-3C-R84S, 3C-TGA, 3C-TGA-Δ5990–6844, 3C-TGA-Δ5990–6974, 3C-TGA-Δ6975–7369, 3C-TGA-Δ7265–7369, 3C-TGA-Δ5990–6844, Δ7265–7369, 3C-TGA-Δ5990–6844 + Δ7220–7369, 3C-TGA-Δ5990–6973 + Δ7265–7369, 3C-TGA-Δ5990–6973 + Δ7220–7369, Δ5993–6844, Δ5993–6973, Δ6974–7369, Δ7265–7369, Δ7220–7369, Δ5993–6844 + Δ7265–7369, Δ5993–6844 + Δ7220–7369, Δ5993–6973 + Δ7265–7369, Δ5993–6973 + Δ7220–7369, 3D-0 aa, 3D-18aa, 3D-37aa, 3D-70 aa, 3D-202 aa, 3D-286 aa, 3D-329 aa, 3D-411 aa, 3D-427 aa):

Indicated mutations were introduced in pPV-Fluc mc with a hammerhead ribozyme at the 5’end of the replicon [67,97] by PCR with primer sets in S1 Table.

### Preparation of lentivirus and PV-non-structural-proteins-expressing cells

HEK293 cells were transfected with the lentivirus expression vectors constructed as above, and packaging plasmids psPAX2 (a gift from Didier Trono, Addgene, 12260) and pVSV-G (Clontech, 631530) using a *Trans*IT-PRO transfection kit (Mirus, MIR 5700). The supernatant of the cells was collected at 48 h and 72 h post-transfection and then mixed before storage at -80°C. HEK293 cells expressing the Tet-On 3G protein were infected with the lentiviruses in the presence of polybrene (4 μg/mL). For the preparation of cells that express capsid proteins of PV1(Mahoney) strain, Tet-AG-PV-3CD(WT) cells that express PV 3CD^pro^ (WT) protein in the presence of DOX [26] were infected with the lentivirus (Tet-AG-PV-3 CD(WT)+PV1-capsid cells).

### RNA transfection

RNA transcripts of PV replicons were obtained using a RiboMAX Express Large Scale RNA Production System (Promega, P1320) with *Dra*I-linearized plasmids of PV replicons. RNA transcripts (0.025 μL) were transfected into the cells (4 × 10^4^ cells per well in 100 μL medium) in a 96-well plate (Corning Incorporated, 3595) using *Trans*IT-mRNA Transfection Kit (Mirus, MIR 2250).

### Preparation of PV pseudovirus (PV_pv_) with a defective PV replicon

Tet-AG-PV-3CD(WT)+PV1-capsid cells (4 × 10^4^ cells per well in 100 μL medium) in a 96-well plate (Corning Incorporated, 3516) were incubated in the presence of DOX (1 mg/L) at 37 **°**C for 20 h. RNA transcripts (0.025 μL) of defective PV replicons were transfected into monolayers of Tet-AG-PV-3CD(WT)+PV1-capsid cells expressing the capsid proteins and the 3CD^pro^ protein. The cells were harvested at 24 h post-transfection of the RNA transcripts and then stored at -20 **°**C. To assess the stability of the genomes, the obtained PV_pv_ was subjected to passage. PV_pv_ solution (10 μL) was inoculated into the DOX-treated Tet-AG-PV-3CD(WT)+PV1-capsid cells (4 × 10^4^ cells per well in 100 μL medium), and then the cells were harvested at day 1 or 2 p.i. Viral RNA was extracted from PV_pv_ solution (100 μL) obtained after two rounds of passage.

### Titration of defective PV_pv_

Tet-AG-PV-3CD(WT) cells (8 × 10^3^ cells per well in 20 μL medium) in a 384-well plate (Greiner Bio-One, 781080) were incubated at 37 °C for 5 h in the presence of DOX (1 mg/L). The cells were inoculated with 5 μL of serially diluted PV_pv_ solution (dilution of 1/1–1/10^5^) and then incubated at 37 °C for 17 h. A 15 μL of the supernatant was removed from each well, and then 10 μL of Steady-Glo Reagent (Promega, E2520) was added to each well. Luciferase signals were measured using a 2030 ARVO X luminometer (Perkin-Elmer). The infectivity of PV_pv_ solution was calculated from the signals in cells infected at an MOI of approximately 0.1.

### Western blot

The cells (8 × 10^5^ cells) were collected in 40 μL of cell lysis buffer (21 mM HEPES buffer [pH 7.4], 0.7 mM disodium hydrogenphosphate, 137 mM NaCl, 4.8 mM KCl, 0.5% Nonidet P-40 and 5 mM EDTA, supplemented with complete-mini protease inhibitor cocktail tablet [Roche, 04 693 159 001]), and then were subjected to e-PAGEL 5–20% gradient polyacrylamide gel electrophoresis (Atto Corporation) in a Laemmli buffer system. Proteins in the gel were transferred to a polyvinylidene difluoride filter (Millipore, Immobilon) and blocked in iBind solution (Thermo Fischer Scientific). Filters were incubated with anti-PV 2C or anti-PV 3A antibodies [36] (rabbit antisera, 1:500 and 1:200 dilution, respectively), then with secondary antibodies (Thermo Scientific, 32460, goat anti-rabbit IgG antibodies conjugated with horseradish peroxidase, 1:200 dilution) in iBind Western System (Thermo Fischer Scientific). Signals were detected with SuperSignal West Femto Maximum Sensitivity Substrate (Thermo Scientific, 34095), then analyzed with ImageQuant 800 (cytiva).

### Whole genome sequence analysis of PV_pv_

cDNAs of PV_pv_ genomes were obtained by reverse transcription-PCR (RT-PCR) using viral RNA as the template using a PrimeScript II High Fidelity One-step RT-PCR Kit (Takara Bio, #R026A) with the 5’ phosphorylated primers EcoRI-S2+ (5- TTAAGAATTCTTAAAACAGCTCTGGGGTTGTACCCACCC-3’) and EcoRI-PV-polyA- (5’- AAAAGAATTCTTTTTTTTTTTTTTTTTTTTTTTTTCTCCGAATTAAAGAAAAATTTACCC-3’). The RT-PCR condition consists of an RT step at 45°C for 15 min followed by inactivation at 94°C for 2 min, 52 cycles of thermal cycling at 98°C for 10 sec, 55°C for 15 sec, and 68°C for 80 sec, and a hold step at 10°C. The RT-PCR products were purified using a NucleoSpin Gel and PCR Clean-up kit (Takara Bio, #740609.250). T4 DNA ligase buffer (NEB, #B0202S) (3 μL) and T4 DNA ligase (NEB, #M0202L) (800 U, 2 μL) were added to the purified RT-PCR products (25 μL). The samples were incubated at room temperature for 2 h for circularization and then purified using a NucleoSpin Gel and PCR Clean-up kit (Takara Bio, #740609.250). Concentration of DNA in the samples was quantified using a Qubit Flex Fluorometer (ThermoFischer SCIENTIFIC) and then adjusted approximately to 50 ng/μL by water. The circularized DNA was analyzed by the nanopore sequencing method (Oxford Nanopore Technologies, SQK-RBK114, https://nanoporetech.com/document/rapid-sequencing-v14-plasmid-sequencing-sqk-rbk114-96) using the Plasmid EZ service (AZENTA) to obtain nearly complete genome sequences. Due to a technical limitation in the base calling of the system, one cytidine in nt 4806–4813 (8 consecutive cytidines) was missing in most of the sequence (42/ 46 samples).

### Structural modeling

Structures of 3AB and 3D^pol^ complex were predicted using AlphaFold 3 server (DeepMind) using default parameters, following the official inference pipeline and model parameters obtained from Google DeepMind under the AlphaFold 3 Terms of Use [98]. The proposed structural models were viewed using The PyMOL Molecular Graphics System (version 3.1.6.1, Schrödinger, LLC) and Chimera [99].

### Statistical analysis

Results of experiments are shown as means with standard deviations. The presented data are representative of at least two independent experiments with two or three biological replicates. Values of *P* < 0.01 by one-tailed *t t*est were considered to indicate a significant difference and were indicated by asterisks (**P < 0.01, ***P < 0.001).

## Supporting information

S1 Data Nucleotide sequence of the genomes of PV_pv_ isolates. (TXT)

S1 Table Plasmids and primers used in this study. (XLSX)

S1 Fig Analysis of the stability of activity of OSBP inhibitors on intracellular OSBP. A PI4KB inhibitor (T-00127-HEV1) or OSBP inhibitors (T-00127-HEV2, -HEV3, or -HEV4) were added to HEK293 cells overexpressing C-terminally EGFP-fused OSBP (OSBP-EGFP). Subcellular localization of OSBP-EGFP was analyzed after 30 min and 18 h treatment.
(TIF)

S2 Fig Infectivity and replication level of PV_pv_ mutants obtained after two rounds of passage. PV isolates that had pseudoreversion are highlighted in red. Ratio of the infectivity (without GuHCl) and ratio of the replication level of PV_pv_ after the passages to those of the original PV_pv_ are shown. The data are representative of two independent experiments with one or two isolates. ND; not detectable.
(TIF)

S3 Fig Schematic view of a pseudorevertant derived from the 3C-TGA-Δ5990–6844 replicon (clone 1). The genome region duplicated by slide-back or non-homologous recombination, possibly during the negative-strand synthesis, is highlighted in red.
(TIF)

S4 Fig Effect of 3C^pro^ provided in *trans* on cleavage of 3AB *in vivo.* Western blot analysis of viral proteins in the cells co-expressing AG-PV-2B2CP3(3D-0 aa) and AG-3C(WT) or AG-3D (WT). The cells were treated with DOX (1 mg/L) for 17 h. Viral proteins were detected by anti-2C, -3A, -3C, or -3D antibodies. The percentage of cleaved 3AB is highlighted in color. The data are representative of three independent experiments with two biological replicates. NS, not significant.
(TIF)

S5 Fig Evaluation of the dominant-negative effect of an inactive 3CD^pro^ variant on the cleavage of 3AB *in vivo.* Western blot analysis of viral proteins in the cells co-expressing AG-PV-2B2CP3(WT) and AG-3CD variants (WT or C147A) or AG-3D(WT). The cells were treated with DOX (1 mg/L) for 17 h. Viral proteins were detected by anti-2C, -3A, or -3D antibodies. The percentage of cleaved 3AB is highlighted in color. The data are representative of three independent experiments with two biological replicates. NS, not significant.
(TIF)

S6 Fig Structural models of PV 3AB and 3D^pol^. Structural models of (A) two molecules of 3AB (highlighted in orange or green) and one molecule of 3D^pol^ (highlighted in purple) or (B) one molecule of 3AB (highlighted in green) and two molecules of 3D^pol^ (highlighted in purple and magenta) were generated by AlphaFold3. The 3B region in 3AB was highlighted in cyan. The aa residues analyzed in this study are highlighted in red, blue, or yellow.
(TIF)

S7 Fig Effect of expression levels of 3CD^pro^ or 3D^pol^ provided in *trans* on cleavage of 3AB *in vivo.* Western blot analysis of viral proteins in the cells co-expressing AG-PV-2B2CP3(3D-0 aa) and AG-3CD(WT) or AG-3D(WT). For moderate or low expression of AG-3CD(WT) or AG-3D(WT), approximately 1/3 or 1/30 of the lentivirus solution, respectively, was used, compared to that used in other experiments. The cells were treated with DOX (1 mg/L) for 17 h. Viral proteins were detected by anti-2C, -3A, or -3D antibodies. The percentage of cleaved 3AB is highlighted in color. The data are representative of two independent experiments with two biological replicates. NS, not significant.
(TIF)
