# Supplementary figures and images for: Poliovirus 3Dpol polymerase region is essential for cleavage of poliovirus 3AB in vivo
Source: PLoS Pathog. 2026 May 13;22(5):e1014241. doi: 10.1371/journal.ppat.1014241 (PMC13186352; doi:10.1371/journal.ppat.1014241)

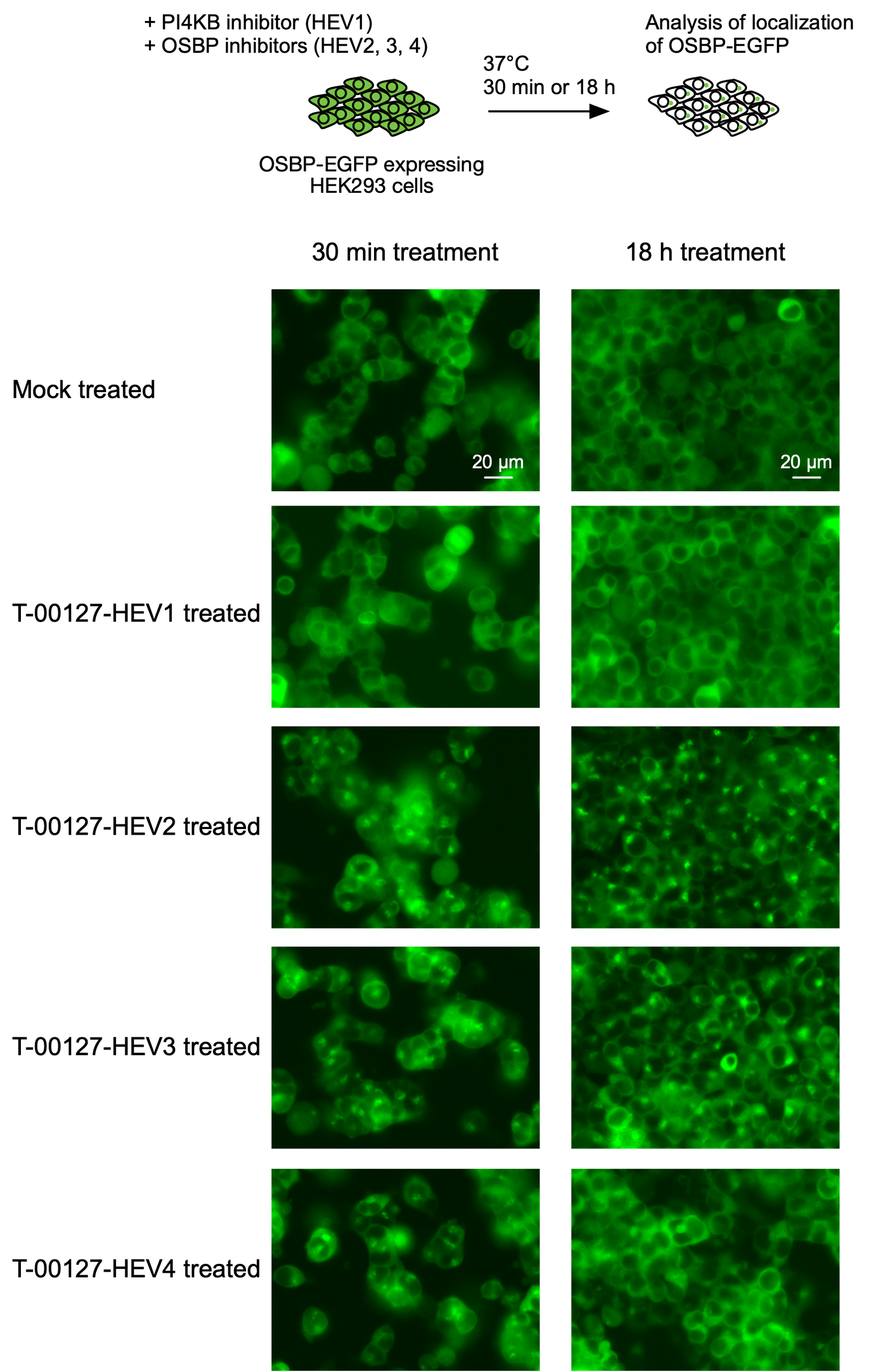

Supplement: S1 Fig — A PI4KB inhibitor (T-00127-HEV1) or OSBP inhibitors (T-00127-HEV2, -HEV3, or -HEV4) were added to HEK293 cells overexpressing C-terminally EGFP-fused OSBP (OSBP-EGFP). Subcellular localization of OSBP-EGFP was analyzed after 30 min and 18 h treatment. (TIF) [file ppat.1014241.s003.tif]

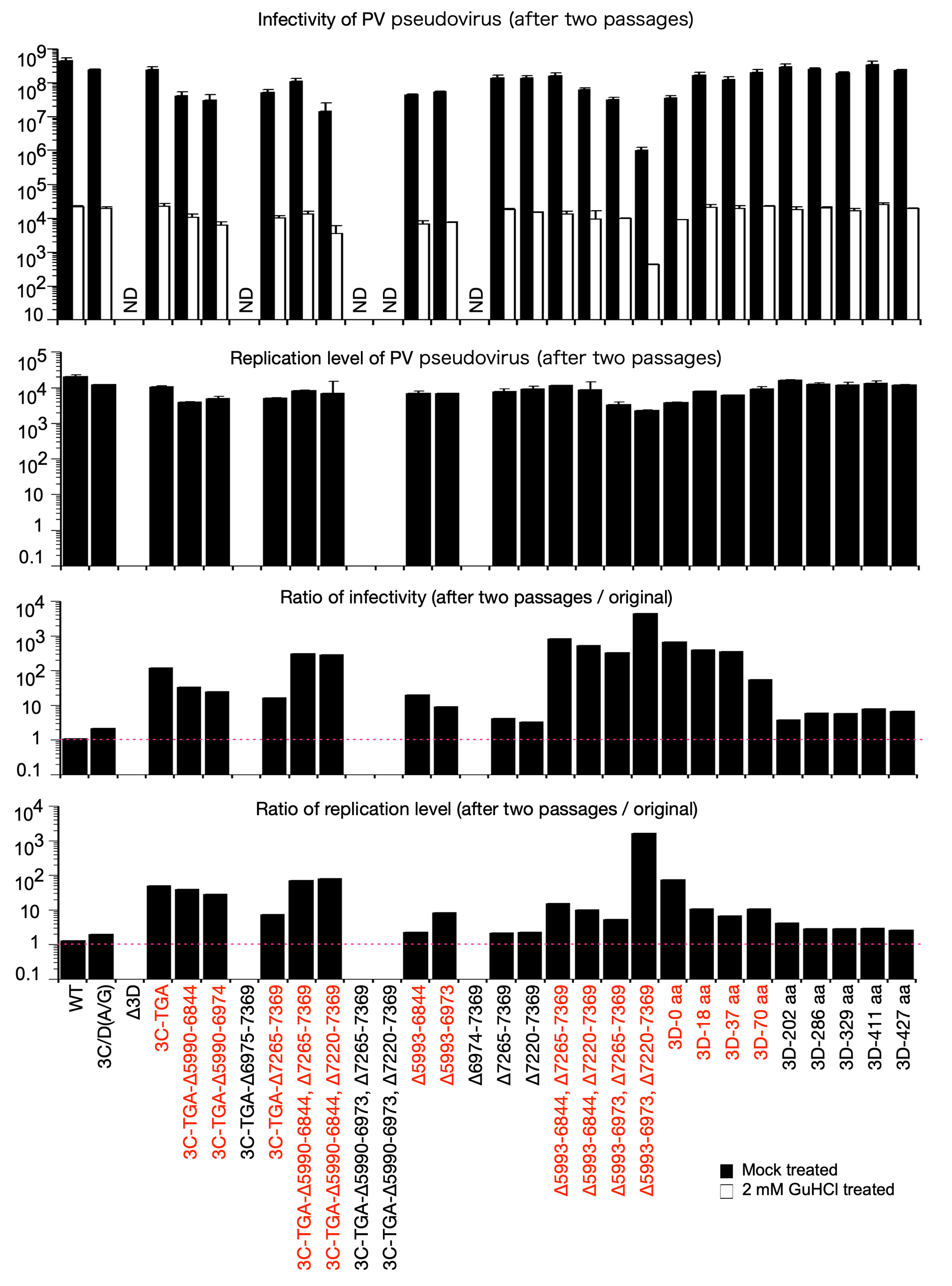

Supplement: S2 Fig — PV isolates that had pseudoreversion are highlighted in red. Ratio of the infectivity (without GuHCl) and ratio of the replication level of PVpv after the passages to those of the original PVpv are shown. The data are representative of two independent experiments with one or two isolates. ND; not detectable. (TIF) [file ppat.1014241.s004.tif]

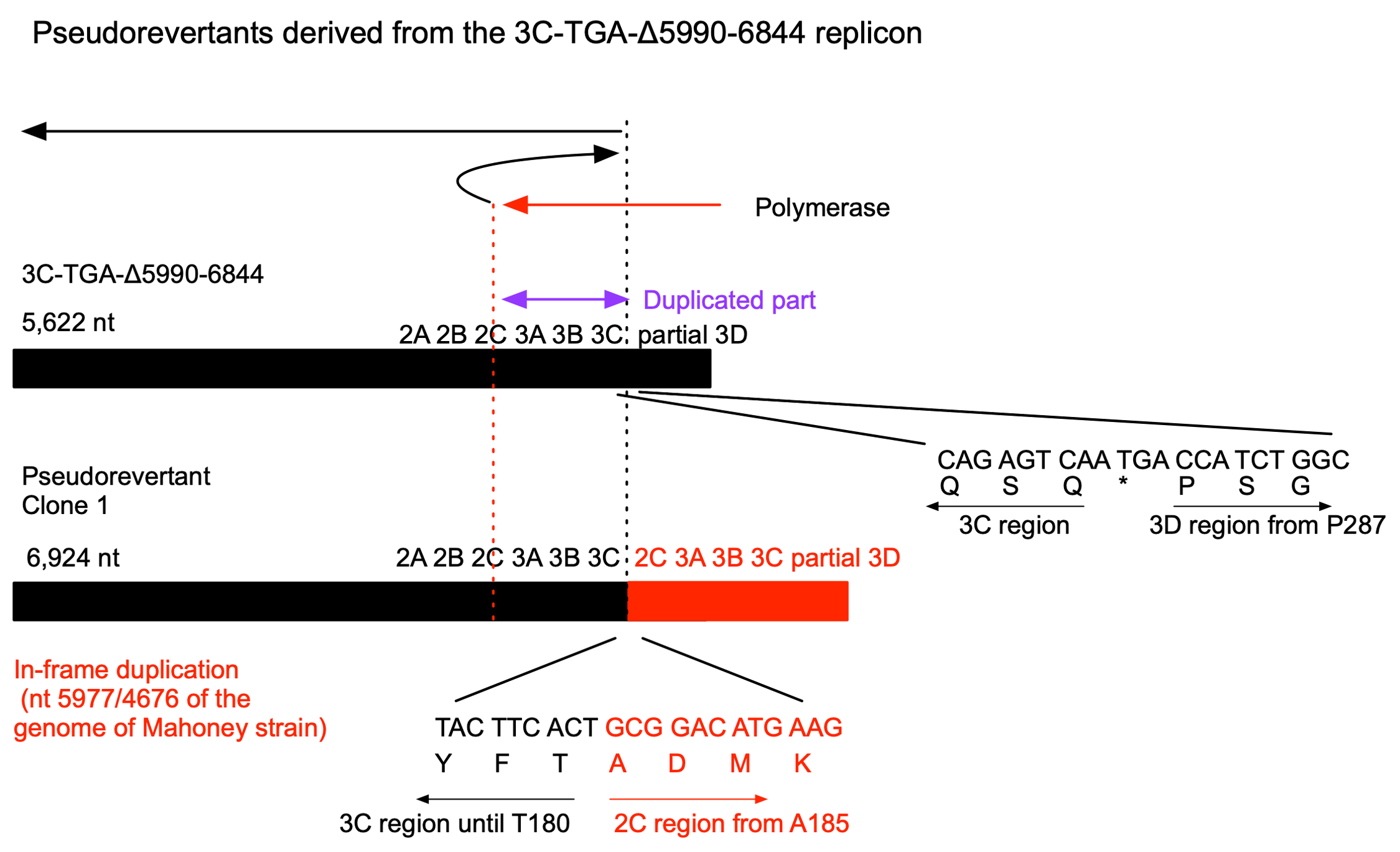

Supplement: S3 Fig — The genome region duplicated by slide-back or non-homologous recombination, possibly during the negative-strand synthesis, is highlighted in red. (TIF) [file ppat.1014241.s005.tif]

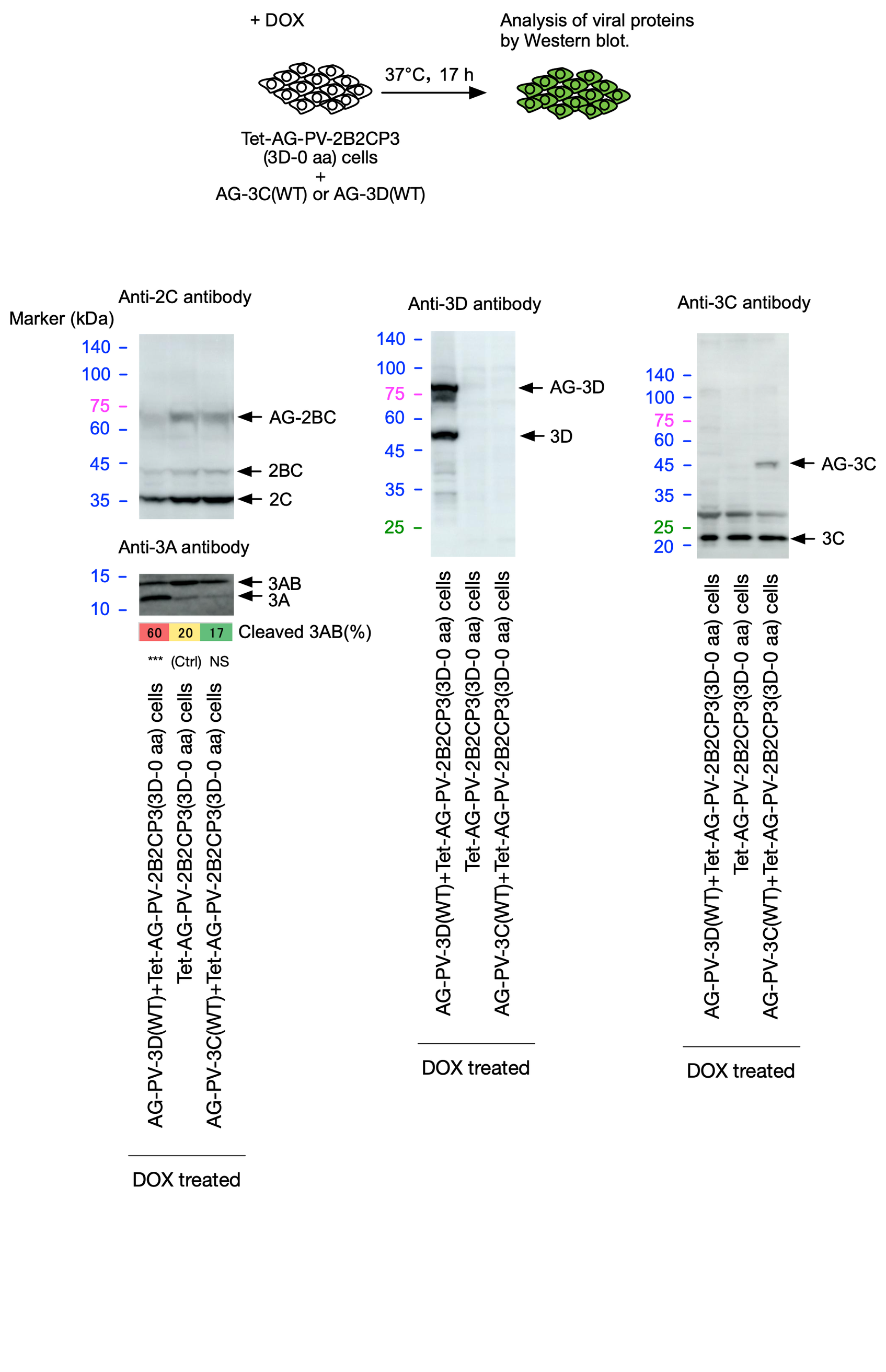

Supplement: S4 Fig — Western blot analysis of viral proteins in the cells co-expressing AG-PV-2B2CP3(3D-0 aa) and AG-3C(WT) or AG-3D (WT). The cells were treated with DOX (1 mg/L) for 17 h. Viral proteins were detected by anti-2C, -3A, -3C, or -3D antibodies. The percentage of cleaved 3AB is highlighted in color. The data are representative of three independent experiments with two biological replicates. NS, not significant. (TIF) [file ppat.1014241.s006.tif]

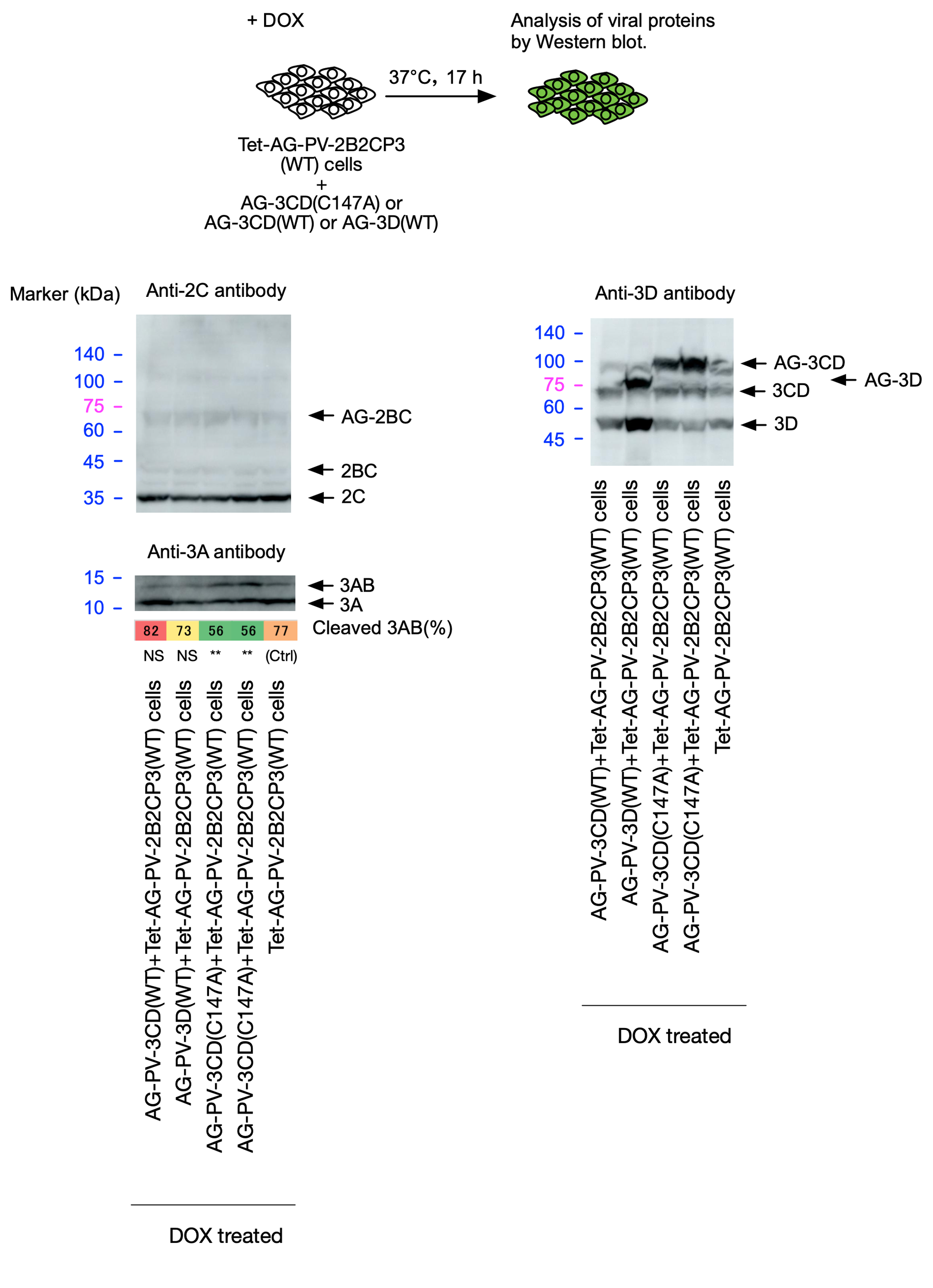

Supplement: S5 Fig — Western blot analysis of viral proteins in the cells co-expressing AG-PV-2B2CP3(WT) and AG-3CD variants (WT or C147A) or AG-3D(WT). The cells were treated with DOX (1 mg/L) for 17 h. Viral proteins were detected by anti-2C, -3A, or -3D antibodies. The percentage of cleaved 3AB is highlighted in color. The data are representative of three independent experiments with two biological replicates. NS, not significant. (TIF) [file ppat.1014241.s007.tif]

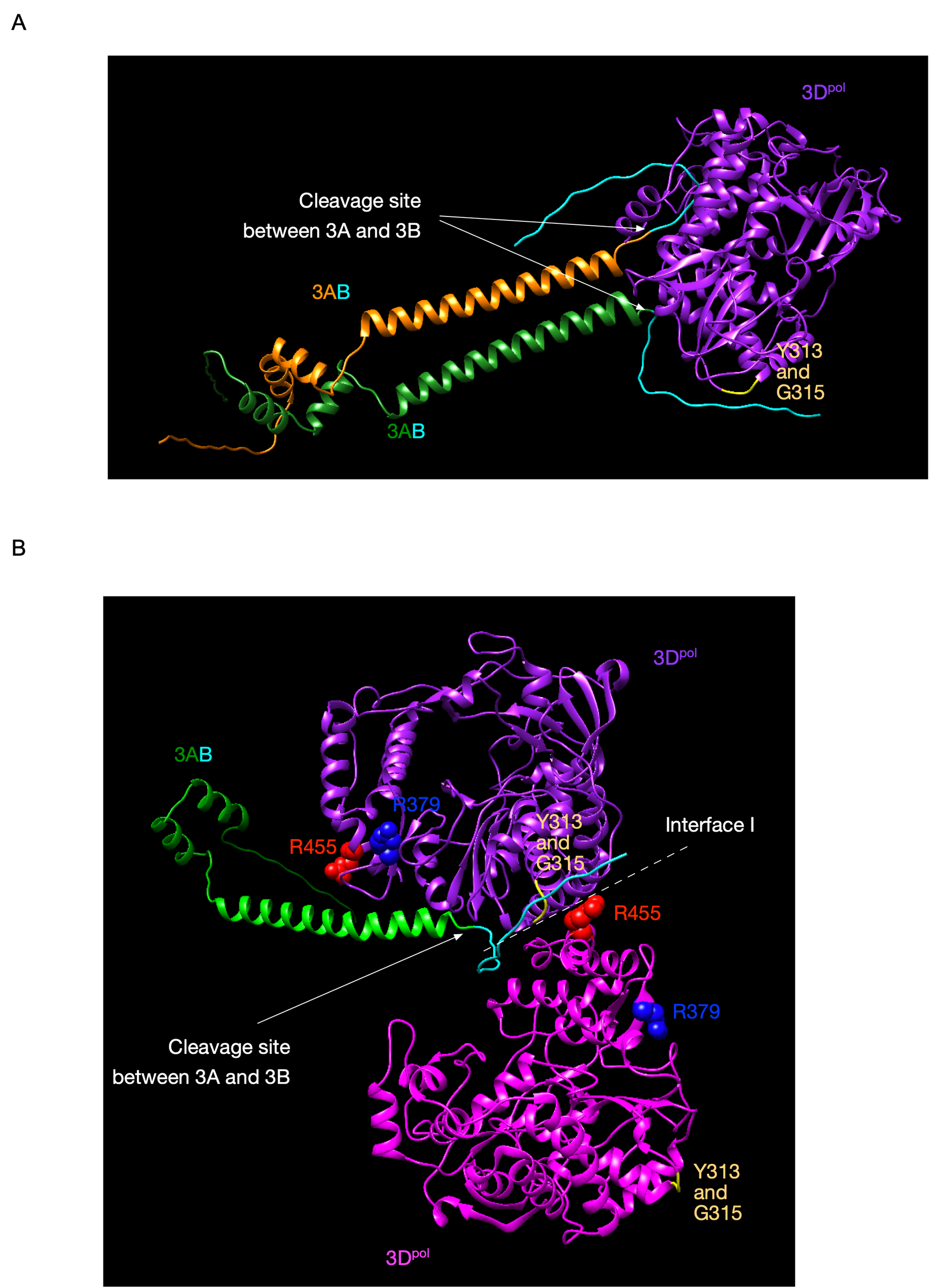

Supplement: S6 Fig — Structural models of (A) two molecules of 3AB (highlighted in orange or green) and one molecule of 3Dpol (highlighted in purple) or (B) one molecule of 3AB (highlighted in green) and two molecules of 3Dpol (highlighted in purple and magenta) were generated by AlphaFold3. The 3B region in 3AB was highlighted in cyan. The aa residues analyzed in this study are highlighted in red, blue, or yellow. (TIF) [file ppat.1014241.s008.tif]

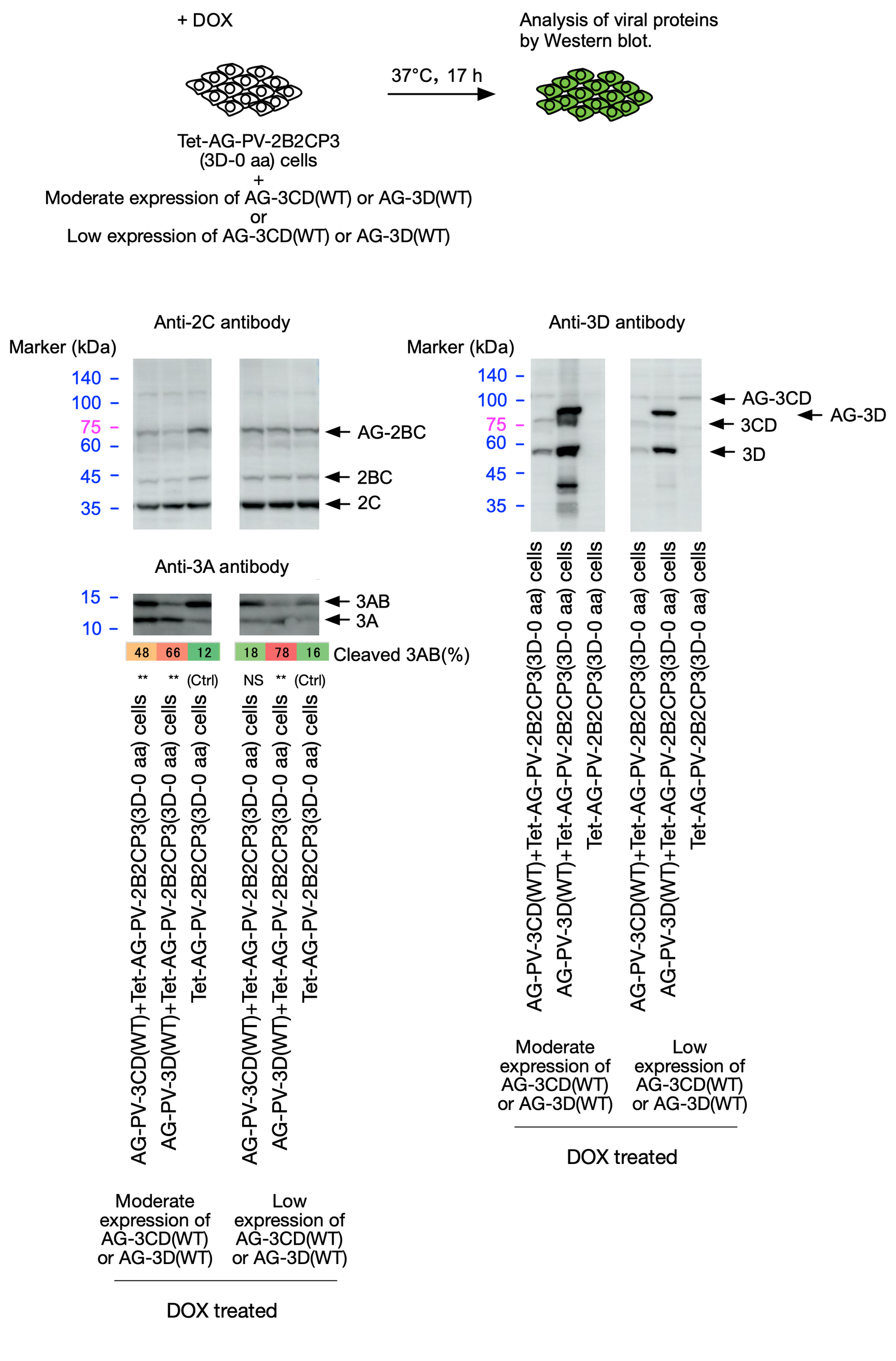

Supplement: S7 Fig — Western blot analysis of viral proteins in the cells co-expressing AG-PV-2B2CP3(3D-0 aa) and AG-3CD(WT) or AG-3D(WT). For moderate or low expression of AG-3CD(WT) or AG-3D(WT), approximately 1/3 or 1/30 of the lentivirus solution, respectively, was used, compared to that used in other experiments. The cells were treated with DOX (1 mg/L) for 17 h. Viral proteins were detected by anti-2C, -3A, or -3D antibodies. The percentage of cleaved 3AB is highlighted in color. The data are representative of two independent experiments with two biological replicates. NS, not significant. (TIF) [file ppat.1014241.s009.tif]
